# Morphologically Different *Pectobacterium brasiliense* Bacteriophages PP99 and PP101: Deacetylation of O-Polysaccharide by the Tail Spike Protein of Phage PP99 Accompanies the Infection

**DOI:** 10.3389/fmicb.2019.03147

**Published:** 2020-01-23

**Authors:** Anna A. Lukianova, Mikhail M. Shneider, Peter V. Evseev, Anna M. Shpirt, Eugenia N. Bugaeva, Anastasia P. Kabanova, Ekaterina A. Obraztsova, Kirill K. Miroshnikov, Sofiya N. Senchenkova, Alexander S. Shashkov, Stepan V. Toschakov, Yuriy A. Knirel, Alexander N. Ignatov, Konstantin A. Miroshnikov

**Affiliations:** ^1^Shemyakin-Ovchinnikov Institute of Bioorganic Chemistry, Russian Academy of Sciences, Moscow, Russia; ^2^Department of Biology, Lomonosov Moscow State University, Moscow, Russia; ^3^Zelinsky Institute of Organic Chemistry, Russian Academy of Sciences, Moscow, Russia; ^4^Research Center “PhytoEngineering” Ltd., Rogachevo, Moscow, Russia; ^5^Winogradsky Institute of Microbiology, Federal Research Center “Fundamentals of Biotechnology”, Russian Academy of Sciences, Moscow, Russia

**Keywords:** bacteriophage, *Pectobacterium brasiliense*, lipopolysaccharide, O-specific polysaccharide, 6-Deoxy-l-talose, random sugar O-acetylation, tail spike, deacetylase

## Abstract

Soft rot caused by numerous species of *Pectobacterium* and *Dickeya* is a serious threat to the world production of potatoes. The application of bacteriophages to combat bacterial infections in medicine, agriculture, and the food industry requires the selection of comprehensively studied lytic phages and the knowledge of their infection mechanism for more rational composition of therapeutic cocktails. We present the study of two bacteriophages, infective for the *Pectobacterium brasiliense* strain F152. *Podoviridae* PP99 is a representative of the genus *Zindervirus*, and *Myoviridae* PP101 belongs to the still unclassified genomic group. The structure of O-polysaccharide of F152 was established by sugar analysis and 1D and 2D NMR spectroscopy:

→ 4)-α-D-Man*p*6Ac-(1→ 2)-α-D-Man*p*-(1→ 3)-β-D-Gal*p*-(1→

$$\begin{array}{c} 3\\ ↑\\ 1\\ \alpha \text{-l-}6\text{dTal}p{\text{Ac}}_{0-2} \end{array}$$

The recombinant tail spike protein of phage PP99, gp55, was shown to deacetylate the side chain talose residue of bacterial O-polysaccharide, thus providing the selective attachment of the phage to the cell surface. Both phages demonstrate lytic behavior, thus being prospective for therapeutic purposes.

## Introduction

*Pectobacterium brasiliense* (Pbr) is a member of the soft rot *Pectobacteriaceae* (SRP) (Adeolu et al., 2016). The representatives of *Pectobacterium* and *Dickeya* genera comprising this family are important pathogens of many crops and ornamental plants worldwide (Ma et al., 2007). Pbr was first identified as a strain group within *Pectobacterium carotovorum*, causing severe black leg disease of potatoes in Brazil (Duarte et al., 2004). This group of strains shared some physiological properties with *P. atrosepticum* (Pat), and was able to grow in a broader range of environmental temperatures. Since then, similar strains were identified as causative agents of black leg and soft rot of potatoes in many countries, including Russia (Malko et al., 2019; Voronina et al., 2019b). Following the taxonomic reassessment of phytopathogenic pectobacteria, Pbr was rated as a subspecies of the *P. carotovorum* (Nabhan et al., 2012), and was recently elevated to the species rank (Portier et al., 2019).

One of the currently applied promising strategies for plant and crop preservations is treating seeds and harvested tubers with bacteriophage preparations. This approach is friendly to the environment and offers a good protection degree against plant bacterial pathogens (Frampton et al., 2012; Svircev et al., 2018). One of the pathogen groups that were studied in this context are pectolytic bacteria *Pectobacterium* and *Dickeya*, where good results of phage application have been reported. Most trials in phage control were applied either to the most widespread or most virulent pathogens–*P. carotovorum* (Lim et al., 2013; Muturi et al., 2019), *P. atrosepticum* (Carstens et al., 2019) and *Dickeya solani* (Adriaenssens et al., 2012; Czajkowski et al., 2017). Most phages suitable for the control of these phytopathogens are members of subfamilies *Autographivirinae, Aglimvirinae*, and lytic phages belonging to the less characterized genera. Usually, such phages are stable and infective in a broad pH range and ionic strength, at temperatures up to 50°C, resistant to chloroform, but are rapidly inactivated by UV irradiation (Gupta et al., 1995; Czajkowski et al., 2015, 2017). Up to now, no bacteriophages specifically infectious to Pbr are reported, to our knowledge. Some phages isolated against *Pectobacterium carotovorum* subsp. *carotovorum* have been shown to infect several strains of Pbr (Lim et al., 2015, 2017; Kim et al., 2019). Information on the factors explaining the host range of particular SRP phages is also limited. Present study is focused on an investigation of Pbr–specific bacteriophages isolated in Russia, and the details of their interaction with the bacterial host on molecular level.

## Materials and Methods

### Bacterial Strain

*Pectobacterium brasiliense* strain F152 (PB29) was isolated in 2014 in a Moscow region from a potato tuber with soft rot symptoms. The strain was analyzed using commercial Biolog GN2 Microplate System (Biolog, Inc.) and API 20E system (bioMérieux). The Biolog GN2 Microplate System and the API 20E system were used in parallel twice, independently. The bacteria were incubated on LB agar (10 g of tryptone, 5 g of yeast extract, and 10 g of NaCl in 1 l, pH 7.2) at 28°C for 24 h prior to analysis. The optical density of the suspension was adjusted as recommended by the manufacturer, and the further procedure was held according to the system instructions. Control for repeatability was held by second round of testing.

### Isolation and Purification of Phages PP99 and PP101

Bacteriophage PP99 and PP101 were isolated in May, 2015, from the same sample of sewage water in a warehouse containing potatoes (Moscow region, Russia). Phages were propagated on a Pbr strain F152 (PB29) in LB at 28°C using a published protocol (Clokie and Kropinski, 2009). The lysate was treated with chloroform, centrifuged to clear the cell debris (8,000 g, 20 min), sterilized by filtering through a 0.22 μm pore size membrane filter (Millipore), and treated with DNase I (0.5 mg/mL, 60 min). Phage particles were pelleted in the ultracentrifuge (100,000 g, 60 min, 4°C, Beckman Type 45 rotor) and further purified by CsCl step gradient (0.5–1.7 g/ml density range) ultracentrifugation (22,000 g, 120 min, 4°C, Beckman SW28 rotor). The resulting suspensions of PP99 and PP101 were dialyzed overnight against suspension medium SM (10 mM Tris HCl, pH 7.4, 10 mM MgSO_4_) to remove CsCl. Purified phages were stored at 4°C in SM buffer as a transparent liquid showing no signs of aggregation.

### Electron Microscopy

Purified phage particles were applied to nitrocellulose/carbon film-coated TEM grids and stained with a 1% uranyl acetate aqueous solution (Ackermann, 2009). The specimens were visualized in a Zeiss Libra 120 electron microscope at 100 kV accelerating voltage. The dimensions of phage virions were averaged from the reads of at least 20 particles.

### Host Range and General Characterization of Phages PP99 and PP101

The host range of phages was tested by standard plaque formation assay using phage serial dilutions and by direct spotting phage suspensions (10^6^ pfu/ml) onto a bacterial lawn. Bacterial strains listed in Table S1 were cultivated on LB agar at 28°C. In phage adsorption experiments, the host strains were grown to an OD_600_ ~0.4 and infected with individual phages at a multiplicity of infection of 0.1, or a mixture of PP99 and PP101 at MOI 0.05 each. Every 1 min after infection, 100 μl aliquots of phage-host mixture were taken and transferred into an 800 μl LB medium supplied with 50 μl of chloroform. After bacterial lysis the mixtures were centrifuged and the supernatant was assayed to determine the amount of non-adsorbed or reversibly bound phages. Phage stability was studied by incubating a 10^7^ pfu/ml phage suspension at different temperatures or in a range of buffer solutions (20 mM Tris HCl/20 mM Na citrate/20 mM Na phosphate), adjusted with NaOH to pH range 4–9. One-step-growth assays were performed according to Adriaenssens et al. (2012). To assay a lytic activity of phages an exponentially growing culture of host bacteria (10^6^ cfu/ml) was mixed with PP99 or PP101 (MOI of 0.1). The mixture was then incubated with shaking at 28°C. Every 10 min, aliquots were taken, chilled, centrifuged, and the appropriate dilutions of the supernatant containing unbound phages were spread on LB agar plates and incubated overnight at 28°C. The next day, colonies were counted. All experiments were performed independently 3–4 times, and the results were averaged.

### Genome Sequencing and Annotation

Bacterial and phage DNA was phenol extracted and fragmented with a Bioruptor Sonicator (Diagenode). Paired-end libraries were constructed using a Nebnext Ultra DNA library prep kit (New England Biolabs) and sequenced on the Illumina MiSeq™ platform (Illumina), using paired 150 bp reads. After filtering with a CLC Genomics Workbench 8.5 (Qiagen), overlapping paired-end library reads were merged with the SeqPrep tool (https://github.com/jstjohn/SeqPrep). Reads from bacteriophages PP99 and PP101 were assembled on a CLC Genomic workbench v. 7.5; reads from a F152 (PB29) bacterial strain were assembled using SPADES 3.6.1 (Bankevich et al., 2012).

The bacterial genome was annotated with the online RAST automated pipeline (http://rast.nmpdr.org/) (Aziz et al., 2008). Phage genomes were annotated by predicting and validating open reading frames (ORFs) using Prodigal 2.6.1 (Hyatt et al., 2010), GeneMarkS 4.3 (Besemer et al., 2001), and Glimmer 3.02 (Delcher et al., 1999). Found ORFs were manually curated to ensure fidelity. Functions were assigned to ORFs using a BLAST search on NCBI databases (http://blast.ncbi.nlm.nih.gov), InterProScan (Mitchell et al., 2015), HHpred (https://toolkit.tuebingen.mpg.de/#/tools/hhpred) (Söding et al., 2005), using databases PDB, SCOP, Pfam, NCBI_CONSERVED. tRNA coding regions were identified with tRNAscan-SE (Schattner et al., 2005) and ARAGORN (Laslett and Canback, 2004). Putative phage promoters were predicted by PHIRE (Lavigne et al., 2004) and phiSITE (Klucar et al., 2009). Resulting genomes were visualized in Geneious Prime, version 2019.2.1 (https://www.geneious.com).

### Genome Comparison and Taxonomy

Bacterial and phage reference genomes were downloaded from NCBI Genbank (ftp://ftp.ncbi.nlm.nih.gov/genbank). The phylogenetic tree of *Pectobacterium brasiliense* was generated by means of a UBCG pipeline, using 92 core genes (Na et al., 2018). To conduct bootstrap analysis phylogeny, we have aligned concatenated sequences of 92 core genes made by UBCG with MAFFT (FFT-NS-x1000, 200 PAM/k = 2), and constructed bootstrap trees with an RAxML program (Stamatakis, 2014) (GTR Gamma I DNA substitution model). The robustness of the trees was assessed by fast bootstrapping (1000).

Genes of phage DNA polymerase, a major capsid protein, RNA polymerase and a terminase large subunit were extracted from downloaded Genbank annotated genomes. Gene products in genomes annotated as “hypothetical protein CDS” were considered as known genes if their pairwise identity with known homologous was more than 50%. Phylograms were generated based on the amino acid sequences of proteins and their concatenated alignments, using Geneious Prime and applying Clustal Omega for sequence alignment with auto settings. Trees were constructed using the maximum likelihood (ML) method with an RAxML program (Stamatakis, 2014) with a GAMMA I BLOSUM62 protein model and the robustness of the trees was assessed by fast bootstrapping (1000). Average nucleotide identity (ANI) was computed using Jspecies (Richter and Rossello-Mora, 2009) (blast algorithm ANIb, 500 bp fragment length for phages and 1020 bp for bacteria). Distance matrix was computed using an Enveomics server (http://enve-omics.ce.gatech.edu) (Konstantinidis and Tiedje, 2005). Digital DNA-DNA hybridization (dDDH) was estimated using a GGDC calculator (https://ggdc.dsmz.de/ggdc.php) (Meier-Kolthoff et al., 2013).

### Tail Spike Protein Cloning, Expression, and Purification

ORF55 of phage PP99 (42,368–44,020 bp range) was PCR-amplified using primers 5′-TATTTCCAGGGCAGCGGATCCGGTTATAGTACAAAGCCAAAAAGA (forward) and 5′-GCTCGAGTGCGGCCGCAAGCTTACAGGTTTGCTGTTACAGAATA (reverse) with generated BamHI and HindIII cloning sites, respectively. The amplified ORF was cloned to vector pTSL (Taylor et al., 2016), using a NEBuilder HiFi DNA Assembly kit (New England Biolabs). Clones carrying the insert were screened by PCR using the same primers, endonuclease hydrolysis, and verified by Sanger sequencing. Protein synthesis was performed in *E. coli* B834(DE3) inducing the expression with 1 mM IPTG at 16°C overnight. Cells were centrifuged at 4,000 g, resuspended in a 20 mM Tris-HCl (pH 8.0), 200 mM NaCl buffer, lysed by ultrasonic treatment (Virsonic, VirTis) and the debris and unbroken cells were removed by centrifugation at 13,000 g. The protein product (PP99 gp55) was purified on a Ni-NTA Sepharose column (GE Healthcare, 5 mL) by 0–200 mM imidazole step gradient in 20 mM TrisHCl (pH 8.0), 200 mM NaCl. The resulting eluate containing the purified protein was dialyzed against 20 mM TrisHCl (pH 8.0) to remove imidazole, and 6× His-tag was removed by TEV protease (12 h at 20°C incubation). The target protein was finally purified on a 5 mL SourceQ 15 (GE Healthcare) using a linear gradient of 0–600 mM NaCl in 20 mM TrisHCl (pH 8.0). Protein concentration was determined spectrophotometrically at 280 nm, using a calculated molar extinction coefficient of 56,435 M^−1^ cm^−1^. The PP99 gp55 oligomeric state was assessed by gel-filtration on a calibrated Superdex 200 10 × 300 column (GE Healthcare).

### Isolation and O-Deacetylation of the O-Polysaccharides

Bacterial lipopolysaccharide (LPS) was isolated in a yield of 8.4% from bacterial cells by the phenol-water method (Westphal and Jann, 1965) and purified by precipitation of nucleic acids and proteins with aq 50% CCl_3_CO_2_H as described (Zych et al., 2001). Then the LPS sample (84 mg) was degraded with aq 2% HOAc for 1.5 h at 100°C. Lipids were removed by centrifugation (13,000 g, 20 min), and the supernatant was applied to the gel-filtration column 70 × 3.0 cm, Sephadex G-50 Superfine (GE Healthcare), using 0.05 M pyridinium acetate pH 4.5 as eluent and monitoring with a differential refractometer (Knauer). A high-molecular mass polysaccharide was obtained in a yield of ~25% of the lipopolysaccharide weight.

Total O-Deacetylation was performed by treatment of an O-polysaccharide sample (21 mg), with 12% aq ammonia (0.6 mL) at 37°C for 16 h. After ammonia evaporation the remaining solution was lyophilized, and an O-deacetylated polysaccharide (DPS) sample was obtained in a yield of 46% of the O-polysaccharide weight.

The effect of the phage PP99 tail spike protein was studied by an addition of a 300 μg aliquote of PP99 gp55 to the O-polysaccharide sample (20 mg), and incubation for 2 h at room temperature. The product was isolated by gel-permeation chromatography, as described above.

### Sugar Analysis

Hydrolysis of the O-polysaccharide was performed with 2 M CF_3_CO_2_H (120°C, 2 h), and the monosaccharides were analyzed by GLC as the alditol acetates (Sawabdekeb et al., 1965) on a Maestro (Agilent 7820) chromatograph (Interlab) equipped with an HP-5ms column (0.32 mm × 30 m), using a temperature program of 160°C (1 min) to 290°C at 7°C min^−1^.

### NMR Spectroscopy

Samples were deuterium-exchanged by freeze-drying from 99.9% D_2_O. NMR spectra were recorded for solutions in 99.95% D_2_O at 30°C on a Bruker Avance II 600 MHz spectrometer with a 5-mm broadband inverse probe head. Sodium 3-(trimethylsilyl) propanoate-2,2,3,3-d_4_ (δ_H_ 0, δ_C_ −1.6) was used as an internal reference for calibration. Two-dimensional NMR spectra were obtained using standard Bruker software, and a Bruker TopSpin 2.1 program was used to acquire and process the NMR data. A spin-lock time of 60 ms and a mixing time of 200 ms were used in two-dimensional TOCSY and ROESY experiments, respectively. A two-dimensional ^1^H, ^13^C HMBC experiment was performed with a 60-ms delay for evolution of long-range couplings in order to optimize the spectrum for coupling constant *J*_H,C_ 8 Hz.

## Results

### Properties and Genomics of Strain F152 (PB29)

**Figure 1:**
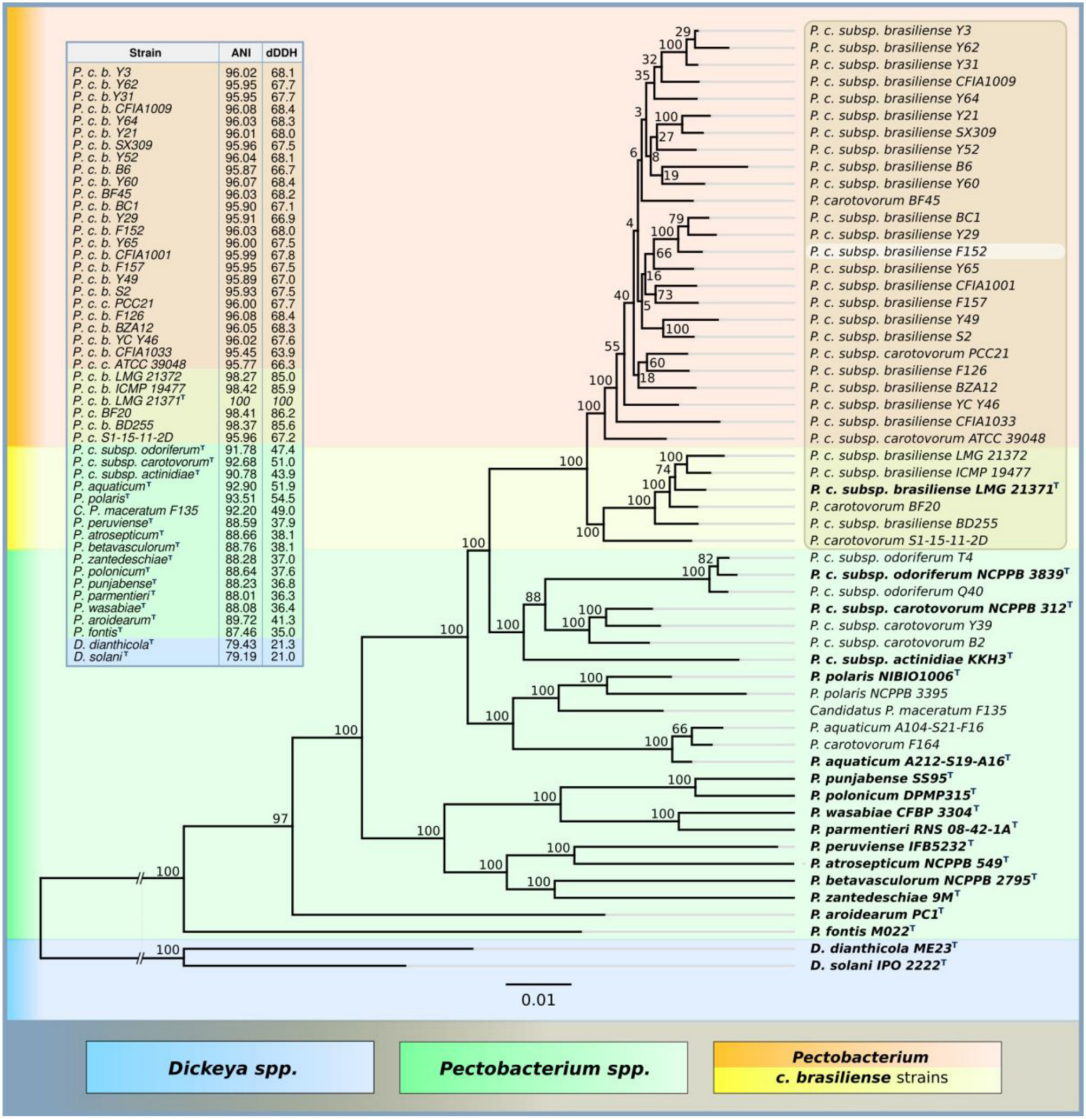
Phylogenetic tree of nucleotide sequences of UBCG proteins of *Pectobacterium* spp. NCBI genomes (RAxML,GAMMA I BLOSSUM62 protein model, with 1,000 bootstrap replicates), ANI and dDDH are compared to *P. brasiliense* LMG 21371 type strain.

*Pectobacterium brasiliense* strain F152 (PB29) was isolated in 2014, and initially was attributed as *P. c*. subsp. *carotovorum*. Further complete genome sequencing identified this strain as a Pbr. Variability in physiology, plant host range, virulence, and genomics of strains representing *P. carotovorum* have promoted the first rough distribution of this genus to separate the subspecies *carotovorum, brasiliense* and *odoriferum* (Nabhan et al., 2012). Later, a number of further separations of *P. carotovorum* subsp. *carotovorum* were offered, forming new species, *polaris* (Dees et al., 2017), *maceratum/versatile* (Shirshikov et al., 2018; Portier et al., 2019), *peruviense* (Waleron et al., 2018), and *aquaticum* (Pédron et al., 2019), based on genomic analysis of available MLST markers, draft and complete genomes in databases. Recently, a substantial redistribution of pectobacterial taxonomy was processed, elevating a number of *P. carotovorum* subspecies to the species level, including Pbr (Portier et al., 2019). Strain F152 follows the general biochemical and physiological properties typical for Pbr species (Nabhan et al., 2012; Portier et al., 2019). Bacterial cells of strain F152 are gram-negative, facultative anaerobes, negative for oxidase, urease, indol production, and gelatin liquefaction. They were negative for acid production from D-arabitol, dulicitol, and sorbitol, and were unable to utilize malonate and citrate. Cells are catalase positive, produce acid from lactose, rhamnose, and trehalose, resistant to 6% NaCl and grow at both 28 and 37°C. Strain F152 induces a hypersensitive reaction in tobacco leaves and causes severe black leg symptoms on green plants and soft rot symptoms on potato tuber disks compared to the characterized strains of *P. polaris, P. aquaticum, P. versatile*, and *P. atrosepticum* in a temperature range from 20 to 28°C. The same properties were observed for other Pbr strains previously isolated in Russia, F126 and F157 (Voronina et al., 2019b). However, the genome sequences of Pbr strains deposited to the NCBI GenBank demonstrate pronounced diversity, forming several branches of the phylogenomic clade (Zhang et al., 2016; Li et al., 2018). Phylogenetic analysis using concatenated sequences of 92 core genes resulted in a similar tree (Figure 1), where the genomes of F126 (RRYQ00000000), F152 (PJDM00000000), and F157 (PJDL00000000) are located in a branch diverged from the Pbr type strain LMG 21371 (=PBR1692). Measurements of ANI and dDDH also demonstrate a genetic difference between two clades of Pbr (Figure 1, Figure S1). Therefore, the properties of strain F152, including phage susceptibility, cannot be directly extrapolated to all strains representing Pbr.

### Phage PP99 and PP101—Biology, Host Range, Morphology

Bacteriophages PP99 and PP101 were isolated using Pbr strain F152 previously found in the same location as a host. Both phages form small (1–3 mm in diameter) plaques, normally indistinguishable from each other. Host ranges of PP99 and PP101 are nearly identical to each other. Both phages infect all strains of Pbr isolated in Central European Russia in 2000–2015, as well as nine of 26 *Pectobacterium* strains available in our collection (Table S1). Besides Pbr, all *Pectobacterium* strains susceptible to PP99 and PP101 belong to the newly formed species *P. versatile* (Pve) (Portier et al., 2019). The only strain that is infected by PP101 but is resistant to PP99 is F100, attributed as Pve by 16S RNA gene sequencing and genomic fingerprinting. Other tested strains belonging to *P. atrosepticum, P. polaris, P. parmentieri, P. carotovorum, P. aquaticum*, some strains of *P. versatile*, and all strains of genus *Dickeya*, were resistant to phages PP99 and PP101.

**Figure 2:**
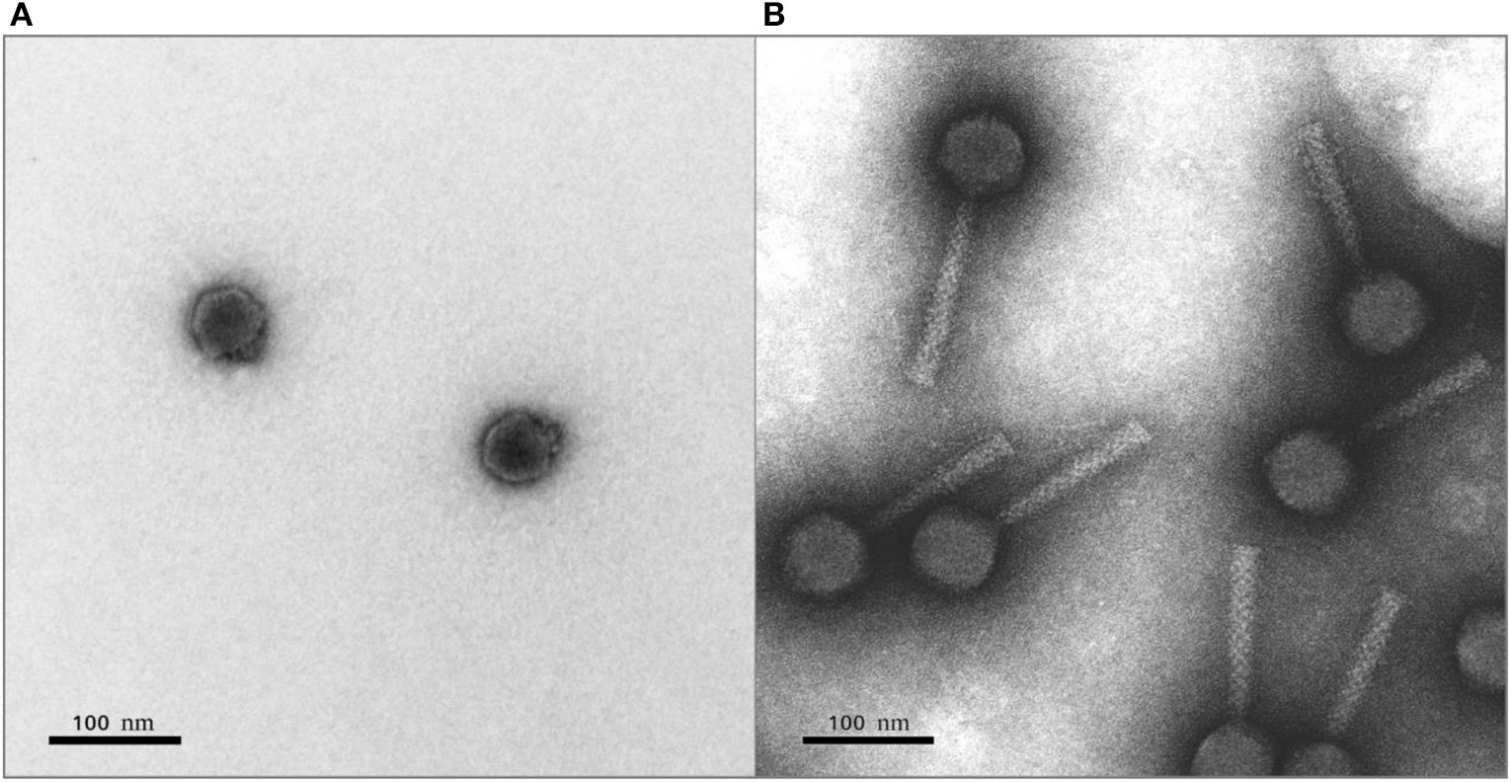
Transmission electron micrograph of bacteriophages PP99 **(A)** and PP101 **(B)**. Staining with 1% uranyl acetate. The scale bar is 100 nm.

The morphology of bacteriophages PP99 and PP101 was assessed using transmission electron microscopy. Phage PP101 belongs to the family *Myoviridae* in the order *Caudovirales*, morphotype A1 (Figure 2B). The tail length is ~132 ± 5 nm, and the head diameter is ~62 ± 3 nm. PP99 shows typical *Podoviridae* morphology (morphotype C1) with an icosahedric capsid of ~54 ± 3 nm in diameter and a short (~10 nm) tail (Figure 2A). According to the proposed unified phage naming (Kropinski et al., 2009), the phages should be referred to as vB_PbrP_PP99 and vB_PbrM_PP101, respectively.

**Figure 3:**
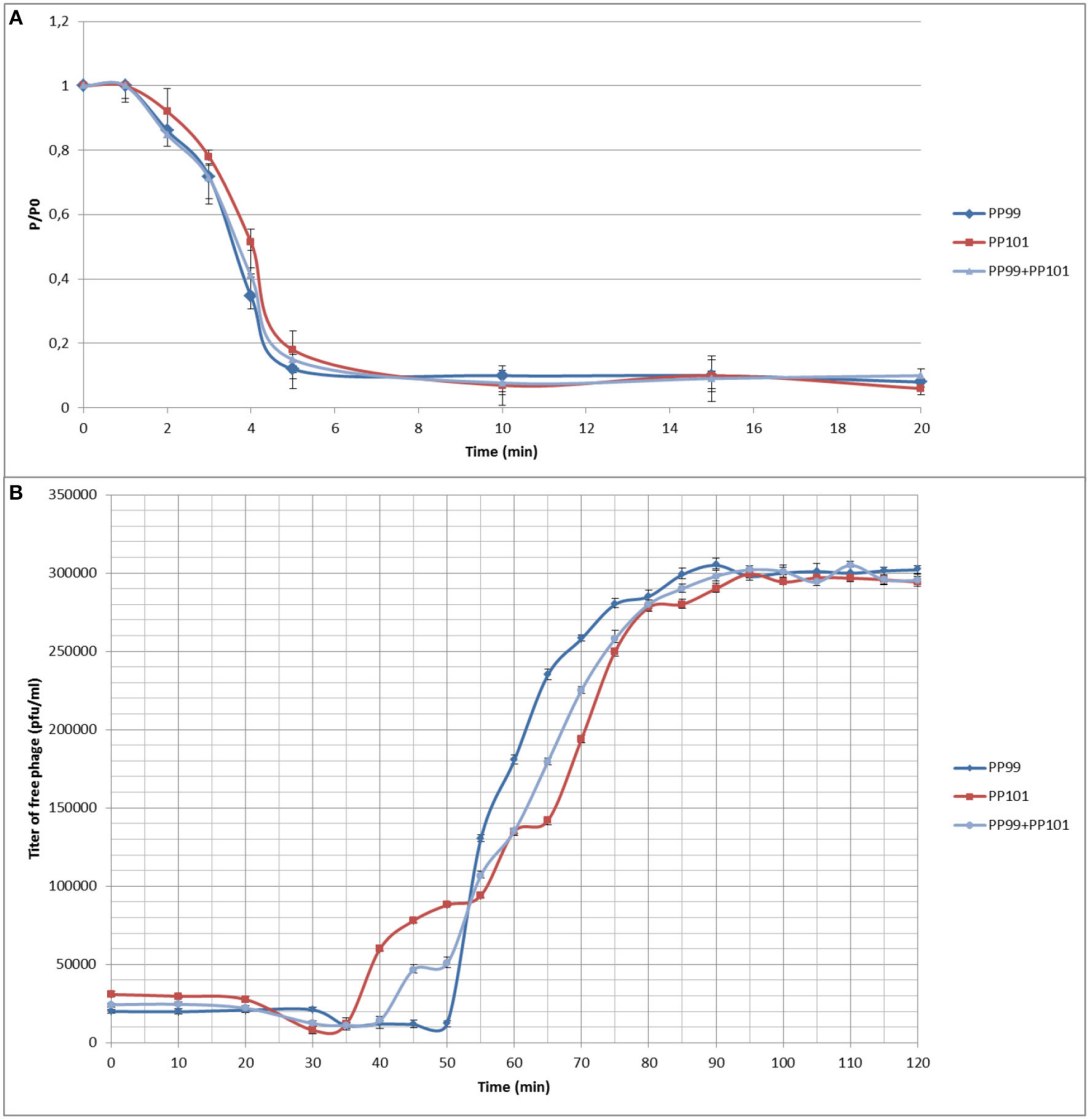
**(A)** Adsorption of phages on the surface of host strain F152: PP99 (blue) and PP101 (red) in MOI = 0.1 separately, and a mixture of PP99 and PP101 (gray) in MOI = 0.05 each. P/Po—ratio of titers of unadsorbed phages to the phage amount initially added; **(B)** One-step growth curves of phages PP99 (blue) and PP101 (red) in MOI = 0.1 separately, and a mixture of PP99 and PP101 (gray) in MOI = 0.05 each.

Both phages demonstrate biological properties close to each other and other studied pectobacterial phages, retaining infectional activity at temperatures below 50°C, and in the pH range 3–8. With respect to their isolation host, Pbr strain F152, phages PP99 and PP101 demonstrate similar infection kinetics adsorbing to bacteria in 4–6 min, with a latent period of 30–35 min and a burst size of 100–150 progeny phages/cell at 28°C (Figure 3). The combined action of PP99 and PP101 did not change the overall shape of the single-step growth curve (Figure 3) in short-term observations. Within a 2 h interval, the concentration of unadsorbed or progeny phages never changed substantially compared to a single-action mode of PP99 or PP101. This indicates an absence of synergistic or antagonistic character of two phages (Schmerer et al., 2014). It may mean that phages PP99 and PP101 independently use the same receptor molecule on the bacterial surface for adsorption, and none of them shows dominant affinity to this receptor; additionally, both phages can overcome the protective systems of Pbr F152 equally effectively.

### Bacteriophage PP99 Genome Features

**Figure 4:**
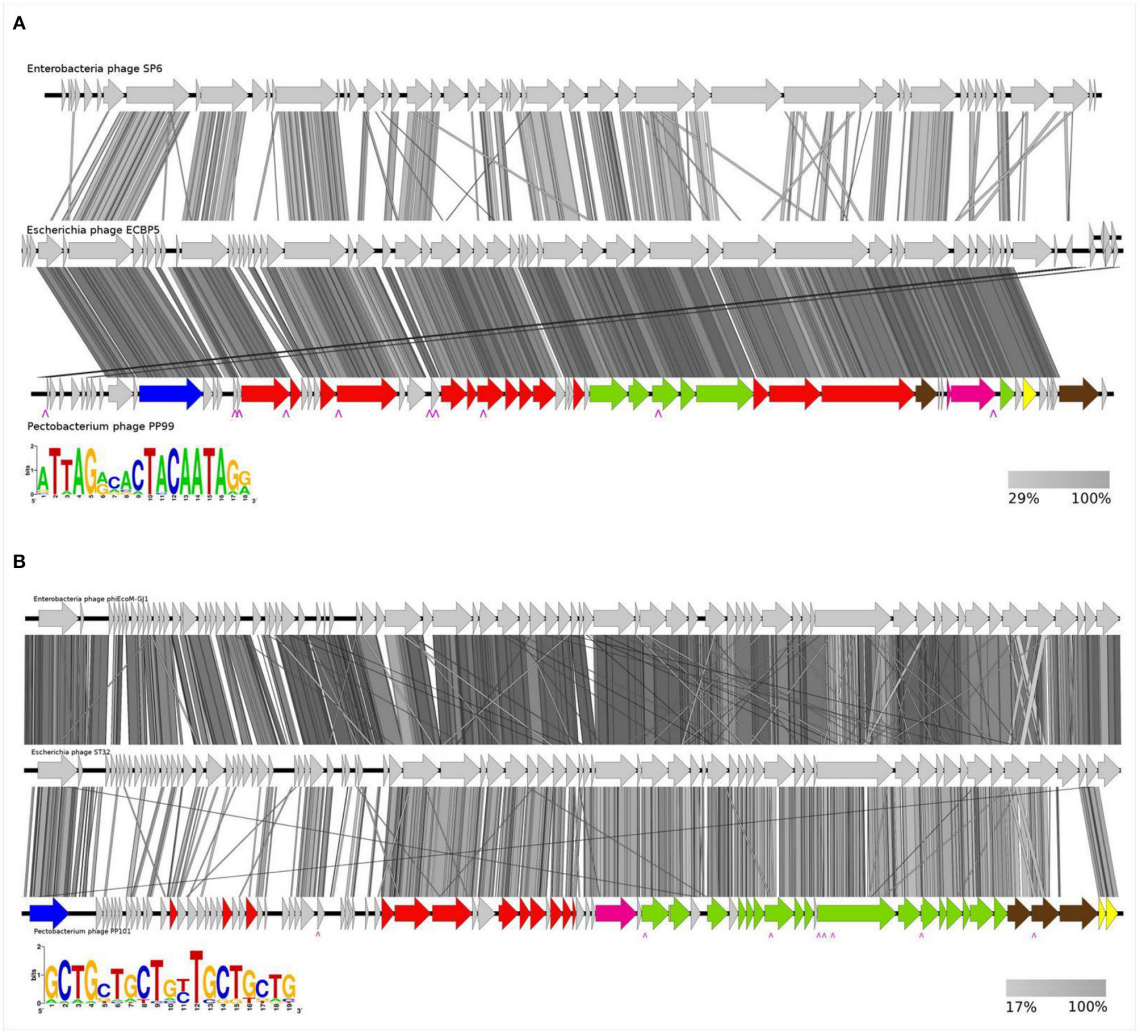
Genome map of bacteriophages. **(A)** PP99, and comparison with phages ECBP5 and SP6; **(B)** PP101, and comparison with PM1 and ST32. Arrows indicate predicted open reading frames presumably responsible for replication and metabolism (red), assembly and packaging (green). ORFs of discussed functions are marked blue (RNA polymerase), magenda (terminase), brown (tail spikes and fibers), and yellow (host lysis). Hypothetical proteins are indicated by tan arrows. The positions of predicted phage promotors are marked. The amino acid sequence similarities between the phages are indicated by gray shading (right insert). The left insert indicates the consensus sequence of the phage promoter.

The genome of bacteriophage PP99 (read in 1006× coverage) consists of 46,609 bp with a GC content of 45.54%. The only bacteriophage with noticeable homology in the genome sequence is the *Escherichia* phage ECBP5 (KJ749827), genetically assessed as a mosaic composition between various *Autographivirinae* phages (Lee et al., 2015; Figure 4A). Phylogenetic trees generated using concatenated alignments of amino acid sequences of DNA polymerase, a major capsid protein and a terminase large subunit show the relationship between PP99 and *Salmonella* phage SP6 (NCBI accession number AY370673) (Dobbins et al., 2004), *Pectobacterium* phage PP1 (NC_019542) (Lee et al., 2012), *Vibrio* phage φA318 (KF322026) (Liu et al., 2014), and a number of other Podoviruses infecting a broad range of bacteria (Figures S2, S3). Thus, PP99 can be assigned as a member of the genus *SP6virus*. This genus is currently named *Zindervirus*, after Norman D. Zinder (1928–2012), who discovered the phage-driven gene transfer. A total of 56 unidirectional ORFs and no tRNA genes were predicted. The functions of 26 ORFs can be predicted as members of transcription/translation, DNA replication/modification and nucleotide metabolism, phage morphogenesis, and host lysis modules. We were able to predict 10 phage-specific promotors (Figure 4A). Twenty-five ORFs were characterized as hypothetical proteins (Table S2). Only three small ORFs can be considered as unique to the PP99 genome. No genes responsible for lysogeny and toxin production were identified. The overall genome layout of PP99 follows the general features of SP6-like phages, including the presence of a pronounced set of early genes preceding the gene-encoding phage RNA polymerase, several apparently non-coding regions, and a holin/muramidase lysis module. Many SP6-like phages, including PP99, share a unified two-component structure of a tail spike (Gebhart et al., 2017; Tu et al., 2017). The gene encoding an adaptor protein providing an attachment of the spike to the tail is separated from the gene encoding the spike in the genome. The C-terminal part of the tail spike protein encoded by PP99 (ORF 55), optimized for an attachment to the surface of the Pbr host, differs from those of other SP6-like phages. The highest (though moderate) sequence similarity is observed with the tail spike protein of phage PP1 infecting a similar pectobacterial host. Sequence analysis of PP99 ORF55 using HHPred predicts a structure resembling (*E*-value 3.3 e^−19^) the tail spike of *Escherichia* phage G7C (*Podovididae, Gamaleyavirus*) with the proposed polysaccharide-modifying enzyme activity (Prokhorov et al., 2017). However, according to the genome analysis, tail spikes of PP99 are not branched as in G7C, so we suggest that PP99 gp55 interacts with the single receptor of Pbr.

### Bacteriophage PP101 Genome Features

Phage PP101 (vB_PbrM-PP101) contains a dsDNA genome (read in 1,458× coverage) of 53,333 bp, with a GC content of 44.94%. We were unable to verify the exact location and size of terminal repeats in the PP101 genome experimentally. However, according to the annotations of similar phages, we consider it as terminally redundant linear with the headful DNA-packing mechanism (Born et al., 2011). Comparative analysis of complete genomes shows noticeable identity (above 80%) of PP101 with *Pectobacterium carotovorum* phage PM1 (NCBI accession number NC_023865) (Lim et al., 2014), *Escherichia* phages ST32 (MF044458) (Liu et al., 2018), phiEcoM-GJ1 (NC_010106) (Jamalludeen et al., 2008), *Erwinia* phages vB_EamM-Y2 (NC_019504) (Born et al., 2011), and Faunus (MH191398) (Table S3). This group of phages forms a separate phylogenetic clade not yet assigned as a taxonomic genus (Figure 4B, Figures S2, S3). Compared to these phages, the genome of phage PP101 has the same unidirectional transcription orientation, and a similar number of identified ORFs organized in functional clusters (Figure 4B). The functions of 36 ORFs could be predicted. Only six ORFs (07, 18, 20, 26, 35, 36), encoding small hypothetical proteins, are unique for PP101. The comprehensive genomic comparison of this group of lytic phages (with the emphasis to *Escherichia* phages) has been presented previously (Liu et al., 2018). We would like to outline a few features of this group of *Enterobacterial* phages, including PP101, that are essential for the realization of the inflectional cycle. First, all phages of this group possess the early gene encoding a single-subunit RNA polymerase, similar to T7-like *Autographivirinae* Podoviruses, and a similar arrangement of transcription-related ORFs. Therefore, we were able to hypothesize the existence of eight consensus promotor sequences throughout the genome that regulates the transcription of gene cascades and key structural genes. The sequence and the proposed positions of phage RNA-polymerase-specific promotors are shown in Figure 4B, and this arrangement provides effective multiplication of the phage in a broad range of conditions and host strains. Second, all the phages of this group contain the two-gene lysis module “holin-SAR endolysin” (ORF 80 and 81 in case of PP101) (Table S3). The third feature is the complex composition of the adsorption apparatus. Three proteins are predicted to form tail fibers of PP101, and these proteins have orthologs in other phages of the group (Table 1). We suggest the presence of the multicomponent tail fibers resembling those of phage T4 (Hyman and van Raaij, 2018) in PP101. PP101 ORF 77 and ORF78 have a high degree of identity with corresponding ORFs of PM1/ST32/phiEcoM-GJ1/vB_EamM-Y2/Faunus, presumably forming the proximal part of the fiber and the knee-knob. A high-resolution EM image of the *Erwinia* phage vB_EamM-Y2 (Born et al., 2011) reveals bicomponent fibers with a globular junction. This observation supports our hypothesis about the structure of PP101 tail fibers involving distal and proximal fibrous parts, and the globular intermediate. HHPred analysis of PP101gp78 shows a number of structural homologs with a β-structural domain responsible for carbohydrate-binding activity (5HON, *E*-value 9.1e-10; 4XJW, 3.9e-9). Being a part of the fiber, this protein may provide irreversible binding to the carbohydrates of the cell surface. The third putative tail fiber component, ORF79, has no homology with any protein of the above phages, except for PM1 infecting *P. carotovorum*. However, genes encoding putative prophage proteins with distantly close (30–40% identity) sequences can be found in the genomes of *Pectobacterium* spp. Therefore, we could propose that the receptor recognized by a similar protein is conservative in *Pectobacteria*, and it was used for host attachment by various phages in an evolutionally long period.

**Table 1:** Genomic features of *Myoviridae* phages similar to PP101.

**Phage**	**NCBI #**	**Host**	**ANI, %**	**Genome size, bp**	**# ORFs**	**Tail fiber 1**	**Tail fiber 2**	**Tail fiber 3**
PP101	KY087898	*Pectobacterium*	100	53,333	81	77	78	79
PM1	NC023865	*Pectobacterium*	93.96	55,098	63	59	60	61
phiEcoM-GJ1	NC010106	*Escherichia*	69.21	52,975	75	70	71	72
ST32	MF044458	*Escherichia*	68.89	53,072	79	74	75	76
Faunus	MH191398	*Erwinia*	68.49	54,065	78	72	73	74
EamM-Y2	HQ728264	*Erwinia*	66.95	56,621	92	82	84	85

### Composition of F152 O-Polysaccharide

**Figure 5:**
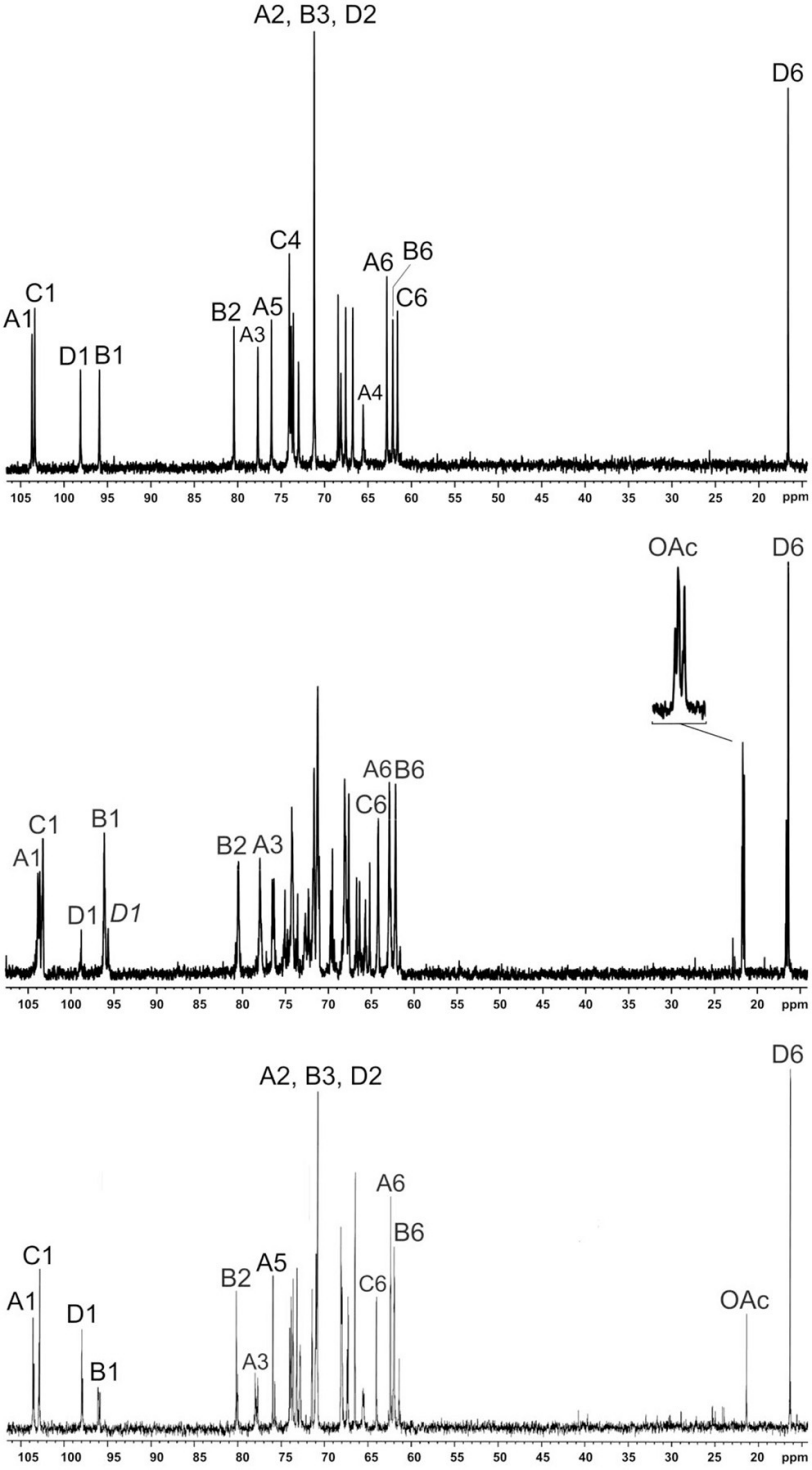
^13^C NMR spectra of F152 O-polysaccharide (middle), O-deacetylated polysaccharide (top), and polysaccharide modified by phage PP99gp55 tail spike protein (bottom). The region for the CO groups is not shown. Numbers refer to carbons in sugar residue denoted by letters, as indicated in Figure 7. *D1* and D1 indicate the 6dTal residues that do and do not include the 2-*O*-acetyl group, respectively.

Sugar analysis of F152 O-polysaccharide (OPS) revealed 6-deoxytalose (6dTalp), mannose, and galactose in the ratios 1–2.2: 1.3 (GLC detector response), respectively. The ^1^H NMR and ^13^C NMR (Figure 5, middle) spectra showed significant structural heterogeneity of the OPS due to non-stoichiometric O-acetylation (there were multiple signals for O-acetyl groups at δ_H_ 2.10–2.23 and δ_C_ 21.5–21.8). The OPS was O-deacetylated with aqueous ammonia to give a regular polymer (DPS). Its ^13^C NMR spectrum (Figure 5, top) contained, inter alia, signals for four anomeric carbons at δ 95.9–103.7, one CH_3_-C group at δ 16.6 (C-6 of 6dTalp), and three HO*C*H_2_-C groups at δ 61.6–62.9 (C-6 of Man and Gal). Accordingly, the ^1^H NMR spectrum of the DPS displayed signals for four anomeric protons at δ 4.44–5.27 and one CH_3_-C group at δ 1.26 (3H, d, *J*_5, 6_ 6.3, H-6 of 6dTal). These data demonstrated that DPS has a tetrasaccharide repeating unit, containing two residues of d-Man and one residue each of d-Gal and l-6dTal.

The D configuration of Man and Gal and the L configuration 6dTalp were established by known regularities in glycosylation effects on ^13^C NMR chemical shifts (Shashkov et al., 1988), combined with calculation of the specific optical rotation of the DPS by Klyne's rule (α_calc_ = 18.9°, α_exp_ = 15.6°).

### Linkage and Sequence Analyses

The ^1^H NMR and ^13^C NMR spectra of the DPS were assigned (Table S4) using one-dimensional ^1^H,^1^H TOCSY, two-dimensional ^1^H,^1^H COSY, TOCSY, ROESY, ^1^H,^13^C HSQC, HMBC, HSQC-NOESY, and HSQC-TOCSY experiments. The configurations of the glycosidic linkages were established by ^13^C NMR chemical shifts of C-5, compared with published data of the corresponding α- and β-pyranosides (Lipkind et al., 1988; Knirel et al., 1992). The β configuration of the Gal residue (unit **A**) was confirmed by a relatively large coupling constant *J*_1, 2_ 7.3 Hz and H-1/H-3 and H-1/H-5 correlations in the ^1^H,^1^H ROESY spectrum of the DPS. The α configuration of the Man and 6dTal residues (units **B**–**D**) was confirmed by H-1/H-2 correlations in the ^1^H,^1^H ROESY spectrum, with no H-1/H-3 and H-1/H-5 correlations.

The two-dimensional ^1^H,^1^H ROESY spectrum showed inter-residue correlations between the following anomeric protons and protons at the linkage carbons: Gal **A** H-1/Man **B** H-4, Man **C** H-1/Man **B** H-2, Man **B** H-1/Gal **A** H-3, and 6dTal **D** H-1/Man **C** H-3 at δ 4.44/3.94, 5.08/4.04, 5.27/3.75, and 5.07/4.02, respectively. These findings were supported by the ^1^H,^13^C HMBC and HSQC-NOESY experiments.

The glycosylation pattern of the monosaccharides was confirmed by low-field positions of the linkage carbons C-3 of unit **A**, C-2 of unit **B**, and C-3 and C-4 of unit **C** at δ 77.7, 80.4, 73.1, and 74.1 in the ^13^C NMR spectrum of the DPS, as compared with their positions at δ 74.1, 72.0, 71.5, and 68.2 in the corresponding non-substituted monosaccharides (Lipkind et al., 1988). The ^1^H and ^13^C NMR chemical shifts of 6dTalp were essentially identical to those for the O-polysaccharide of *Aeromonas hydrophila* O:34, in which this monosaccharide terminated a side chain (Knirel et al., 2002).

**Figure 6:**
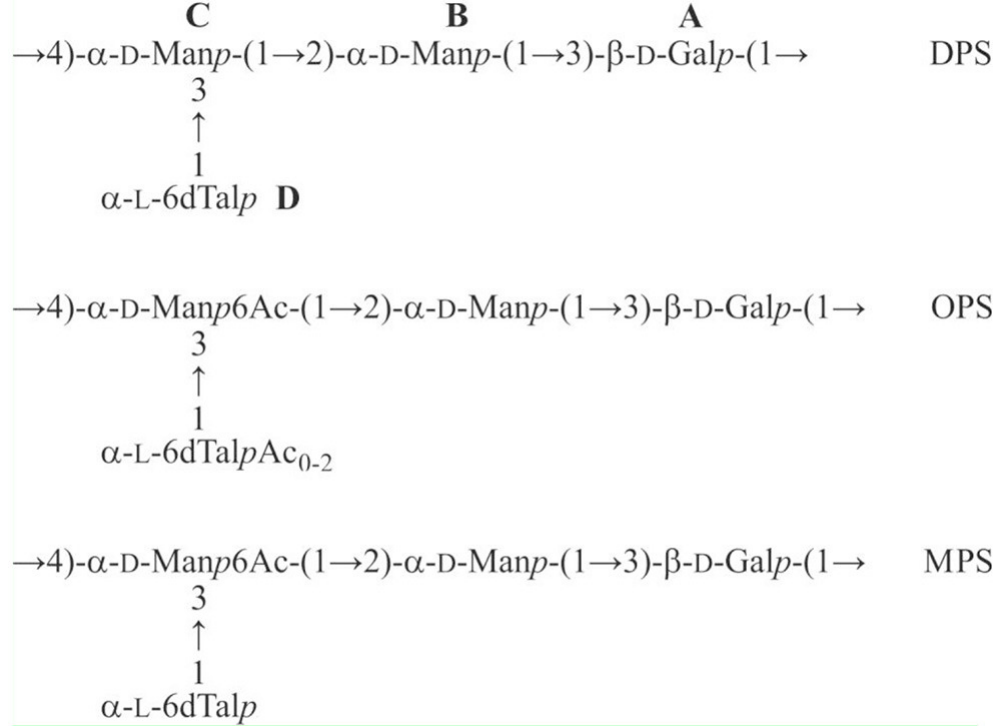
Structures of the O-polysaccharide (OPS), O-deacetylated polysaccharide (DPS), and modified polysaccharide (MPS) of *Pectobacterium brasiliense* strain F152.

Based on the data obtained, we conclude that the DPS has the structure shown in Figure 6, middle.

### O-Acetylation Pattern of the O-Polysaccharide

A comparison of the 1D and 2D NMR spectra of the initial OPS and DPS showed that the signals for units **A** and **B** have almost the same ^1^H and ^13^C NMR chemical shifts in both samples, and hence, they are not O-acetylated in the OPS. In the ^1^H, ^13^C HSQC spectrum of the OPS, a major part of the H-6/C-6 cross-peak of d-Man **C** shifted downfield to δ_H_/δ_C_ 4.35, 4.53/64.2 from its position at δ_H_/δ_C_ 3.81, 3.96/61.6 in the spectrum of DPS. Accordingly, the signal for C-5 of d-Man **C** shifted upfield from δ 73.1 in the DPS to δ 71.9 in the OPS (a β-effect of O-acetylation). As judged by the ratio of integral intensities of the signals for the O-acetylated and non-acetylated forms of Man **C**, the degree of O-acetylation of this sugar residue at position 6 is ~75%.

**Figure 7:**
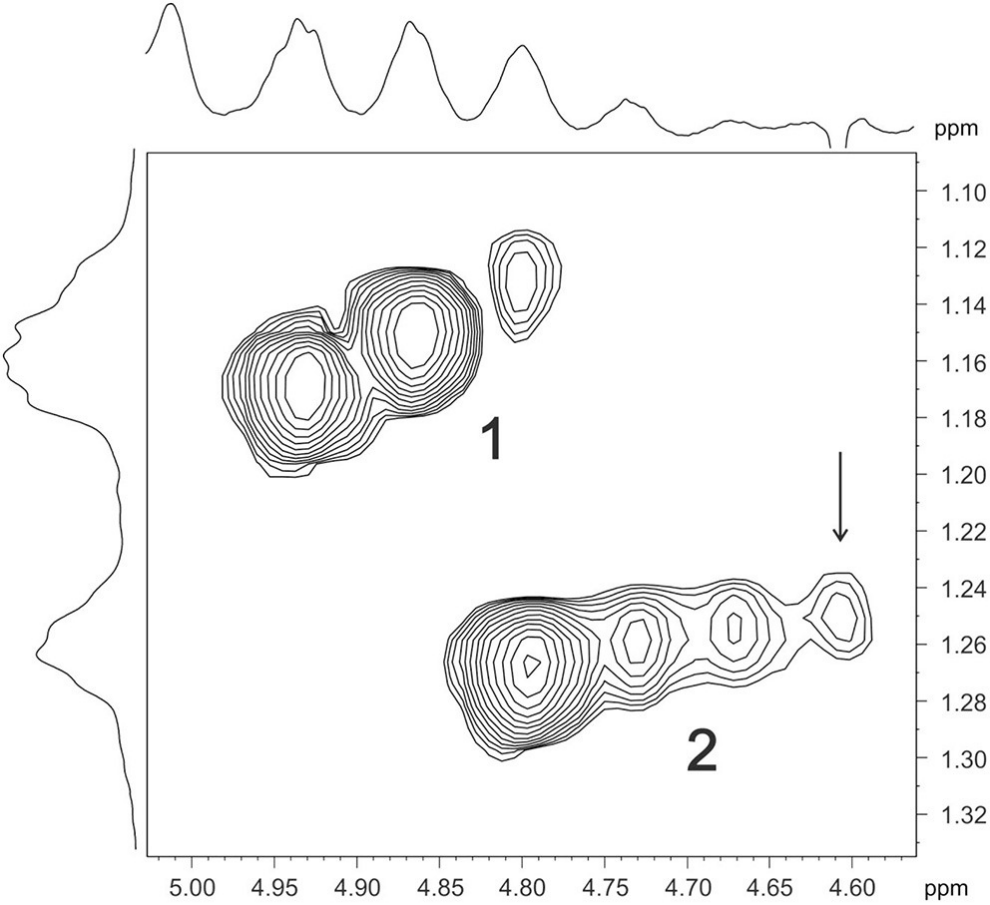
Part of a 600-MHz two-dimensional COSY spectrum of F152 O-polysaccharide, displaying 6dTal H-5/H-6 correlations. Two series of cross-peaks (1 and 2) were assigned to the O-acetylated forms of 6dTal that do and do not include the 4-*O*-acetyl group, respectively. Arrow points to the H-5/H-6 cross-peak of a non-acetylated 6dTal residue.

The 6dTal residue showed multiple signals in the NMR spectra of the OPS, due to the presence of various O-acetylated forms. Particularly, in the 2D ^1^H, ^1^H COSY spectrum, there were seven H-5/H-6 cross-peaks for 6dTalp, which formed two series, 1 and 2 (Figure 7). Such an O-acetylation pattern is similar to that reported for the O-polysaccharide of *Aeromonas hydrophila* O:34 (Knirel et al., 2002). Series 1 of four cross-peaks contained the H-5/H-6 cross-peak for the non-acetylated form at δ 4.61/1.25 (in Figure 5, this cross-peak is indicated by an arrow; compare with the 6dTalp H-5/H-6 cross-peak at δ 4.62/1.26 in the COSY spectrum of the DPS). As the H-5 and H-6 chemical shift are influenced mostly by an acetyl group at O-4, the three other peaks of Series 1 were assigned to the O-acetylated forms that do not include the 4-O-acetyl group, namely to the 2-O-acetylated, 3-O-acetylated, and 2,3-di-O-acetylated forms (Knirel et al., 2002). Correspondingly, the three cross-peaks of series 2 were assigned to the 4-O-acetylated, 2,4-di-O-acetylated and 3,4-di-O-acetylated forms of 6dTal. Therefore, the O-polysaccharide of Pbr F152 has the structure shown in Figure 6, top.

### Properties of Recombinant PP99 Tail Spike Protein Gp55, and Modification of the O-Polysaccharide

Highly copious moieties such as cell surface polysaccharides, including the O-antigens of lipopolysaccharides (LPS), are good candidates for receptor interaction with phages (Steinbacher et al., 1996). Tail spikes contain processive enzymatic domains (Leiman and Molineux, 2008) that depolymerize (Barbirz et al., 2008) or deacetylate (Prokhorov et al., 2017) OPS to allow a phage to attach to the cell surface. Recombinant tail spike proteins of phages were shown to feature mostly uniform trimeric β-helical architecture (Fokine and Rossmann, 2014). The enzymatically active domains located either on the surface of the resulting trimeric prism, or within the secondary structure elements such as loops protruding from the prism (Leiman and Molineux, 2008). Recombinant PP99 gp55 tail spike protein forms trimeric complexes spontaneously (Figure S4), and is easily purified to homogeneous state. The protein tends to aggregate at high concentrations, so the structural research is hindered. In a dilute solution (<2 mg/mL), PP99 gp55 is stable for a few weeks at 4°C. Treatment of the O-polysaccharide with a PP99 tail spike protein resulted in a modified polysaccharide (MPS), which was studied by NMR spectroscopy, as described above for the initial polysaccharide (for the 13C NMR spectrum of the MPS, see Figure 5, bottom). The H-6/C-6 and H-5/C-5 cross-peaks of the 6-O-acetylated d-Man **C** residue in the ^1^H,^13^C HSQC spectrum of the MPS were found at the same positions as in the OPS. In contrast, signals of the variously O-acetylated l-6dTal **D** residues were absent from the MPS, and the cross-peaks of α-l-6dTal were at the same positions as in the DPS. Therefore, the MPS has the structure shown in Figure 6, bottom.

## Discussion

Soft rot and black leg caused by *Pectobacterium* and *Dickeya* spp. are responsible for considerable damage to the potatoes, both during the vegetation season and post-harvest storage (Pérombelon, 2002). No effective control methods currently exist to combat these diseases. Considering the limitations in using pesticides and antibacterial compounds, especially post-harvest, there exists a noticeable demand for new safe and environmentally friendly alternative approaches to pathogen control. The use of bacteriophages to combat plant pathogenic bacteria is one of such promising methods. Phages naturally occur in the environment and are easily biodegradable, which make them suitable for organic agriculture (Frampton et al., 2012; Buttimer et al., 2017; Svircev et al., 2018). Successful applications of phages to protect potatoes from SRP are reported in simulated conditions (Czajkowski et al., 2017; Carstens et al., 2019) and field trials (Adriaenssens et al., 2012; Voronina et al., 2019a). However, these experiments employed a limited number of phages and/or model strains of pathogen initially known to be susceptible to the phage used. An implication of phage control to real horticulture needs to consider many additional factors, including the diversity of pathogens (Svircev et al., 2018).

**Figure 8:**
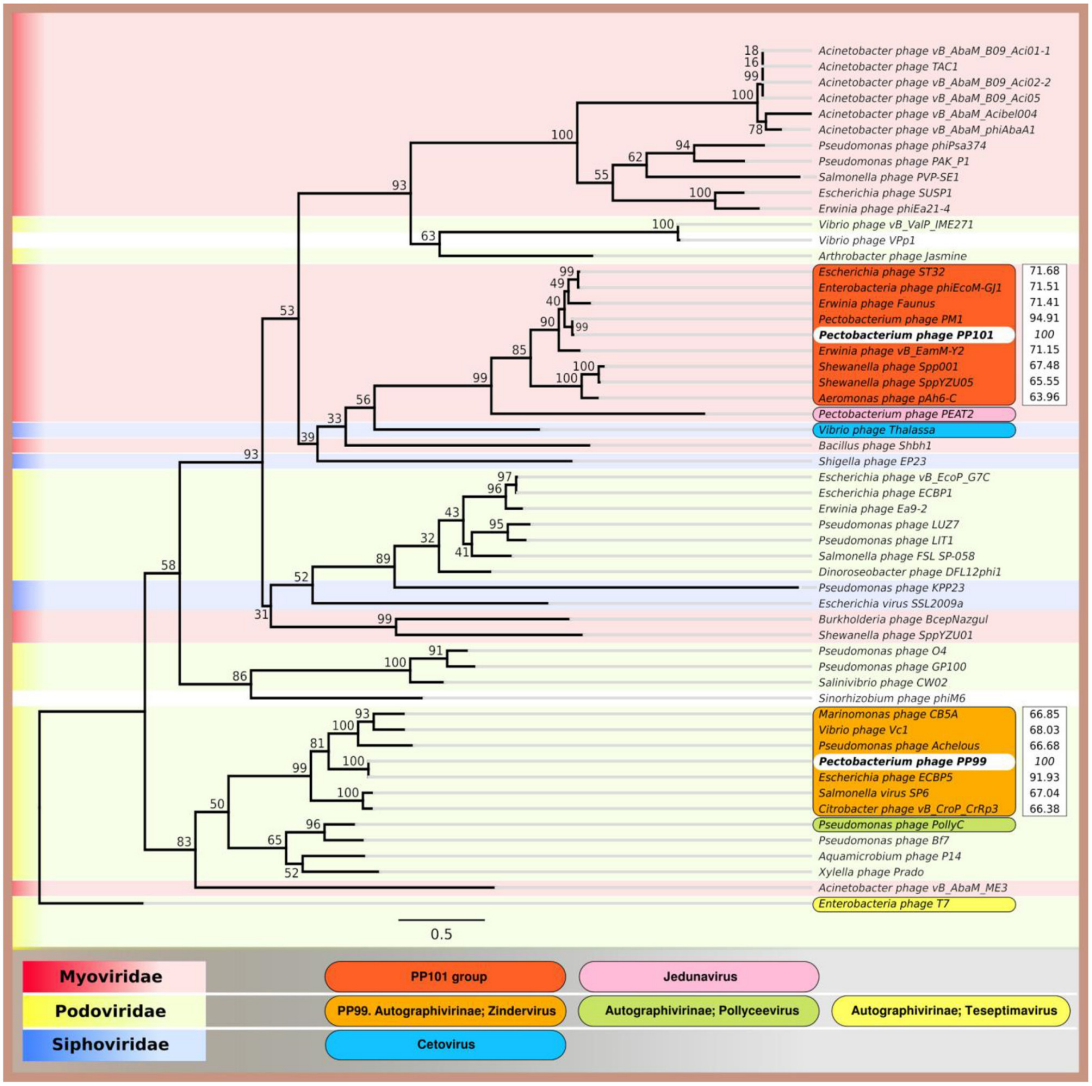
Phylogenetic tree of amino acid sequences of the major capsid proteins of *Pectobacterium brasiliense* phage PP99, *Pectobacterium brasiliense* phage PP101, and 53 phages using RAxML (GAMMA I BLOSSUM62 protein model with 1,000 bootstrap replicates), and ANI compared to PP99 and PP101 calculated with Jspecies.

In this paper we investigate two different phages, isolated using *Pectobacterium brasiliense* as a major host. Pbr has been shown to play an important role in potato soft rot pathogenesis in many countries worldwide in recent years. However, phages specifically infecting this bacterium are understudied, as are the details of their interaction with the host. Both studied phages, PP99 (vB_PbrP_PP99) and PP101 (vB_PbrM_PP101), are lytic phages suitable for phage control. These phages demonstrate different morphology, and can be phylogenetically assigned. PP99 belongs to the genus *Zindervirus* of the *Autographivirinae* subfamily, and PP101 is a member of a clade of unclassified *Myoviridae* phages infecting *Enterobacteria* (Jamalludeen et al., 2008; Lim et al., 2014; Liu et al., 2018) that can be potentially assigned as a new genus of bacteriophages (Table 1, Figure 8). It is worth noting that despite the morphologic difference of PP99 and PP101, their genomes share some features common for “T7-like phages”: unidirectional assignment of ORFs, clear distribution of ORFs to separate gene cascades, early position of the gene encoding the single-subunit RNA polymerase, and a similar arrangement of promotors presumably specific to this polymerase. Some of such features were noticed earlier for phages similar to PP101 (Jamalludeen et al., 2008; Liu et al., 2018). Some evolutionary relationship between phages PP99 and PP101 may be traced based on the phylogeny of DNA polymerase, the terminase large subunit, and, to a lesser extent, major capsid protein genes (Figure 8, Figures S2, S3).

Phages PP99 and PP101 demonstrate similar infection parameters with respect to their type host, Pbr strain F152 (PB29). Host range of tailed phages is largely defined by interaction of their baseplate structures (tail fibers and tail spikes) with molecules on the bacterial surface (Fokine and Rossmann, 2014), as earlier papers suggest. Many bacteriophages use tail spike/fiber proteins for primary contact with bacterial surface polysaccharides. A recognition mechanism involving lipopolysaccharides (LPS) and capsule polysaccharides was suggested as common for SPR phages (Evans et al., 2010) and was experimentally shown for *Dickeya* phage PP35 (Kabanova et al., 2019) and *Pectobacterium carotovorum* subsp. *carotovorum* phage POP72 (Kim et al., 2019). Therefore, our further investigation was directed to reveal the molecular details of phage-host interaction. We have determined the structure of O-polysaccharide of F152, which has no direct analogs in the bacterial world, especially in its acetylation pattern, with ~75% repeating units of d-mannose residues in the main chain O-acetylated at position 6, and ~90% side-chain 6-deoxy-l-talose residues is randomly mono- or di-O-acetylated at any position (Figure 6). This unique composition of F152 O-polysaccharide requires very specific arrangement of amino acid residues on the surface of the proteins of phage recognition apparatus to provide selective contact with the bacterial host. The adsorption system of phage PP101 involves three putative tail fiber proteins (ORF 77–79). This composition is conserved in all phages similar to PP101 (Table 1), and the product of ORF79 is believed to be responsible for the contact with the receptor. However, we were unable to obtain functionally active PP101gp79, therefore this hypothesis still requires experimental proof.

Unlike tail fibers, the structural architecture of phage tail spike proteins performing the contact with the receptor and often bearing the enzyme domain degrading or modifying bacterial polysaccharide is relatively unified (Leiman et al., 2007; Leiman and Molineux, 2008). The genome of phage PP99 contains the gene encoding such tail spike proteins. The recombinant product of this gene, PP99gp55, forms a trimeric biologically active protein that deacetylates the carbohydrate chain of Pbr OPS. The removal of the acetyl group occurs at the side chain of 6-deoxytalose residue, but the acetylated mannose residue of the main chain remains unchanged. We could suggest that the removal of the acetyl provides the spatial flexibility of sugar chains sufficient for the penetration of the tail spike protein to the cell surface. Similar dependence of phage specificity upon the modification pattern of bacterial OPS was previously observed for *Escherichia* phages (Bertozzi Silva et al., 2016; Letarov and Kulikov, 2017).

The knowledge of phage adsorption mechanism and cell receptors used in the course of phage infection is beneficial for the construction of phage cocktails to combat bacterial infections in medicine, agriculture, and the food industry. The use of phages employing different cell receptors of the same bacterial host usually expands an efficacy of antibacterial action, and reduces the possibility of the evolution of phage-resistant mutants. In the studied case, *Pectobacterium brasiliense* phages PP99 and PP101 provide no advantage for combined use, but either of them can be potentially included in phage preparations to control the soft rot of potatoes.

## Data Availability Statement

The complete genome sequences of phages PP99 and PP101 have been deposited in the NCBI database under the GenBank accession numbers KY250034 and KY087898, correspondingly. Draft genome assembly of strain F152 (PB29) and related information can be found in the NCBI database under the GenBank Accession number PJDM00000000.1.

## Author Contributions

MS, AI, and KAM designed the experiment. AL, MS, AMS, EB, AK, EO, SS, and ASS planned and performed experiments. AL, PE, KKM, ST, AI, YK, and KAM performed data analysis. AL, AI, and KAM wrote the manuscript.

### Conflict of Interest

EB, AK, and AI are employed by Research Center “PhytoEngineering” Ltd. The remaining authors declare that the research was conducted in the absence of any commercial or financial relationships that could be construed as a potential conflict of interest.
