# Supplementary figures and images for: Morphologically Different Pectobacterium brasiliense Bacteriophages PP99 and PP101: Deacetylation of O-Polysaccharide by the Tail Spike Protein of Phage PP99 Accompanies the Infection
Source: Front Microbiol. 2020 Jan 23;10:3147. doi: 10.3389/fmicb.2019.03147 (PMC6989608; doi:10.3389/fmicb.2019.03147)

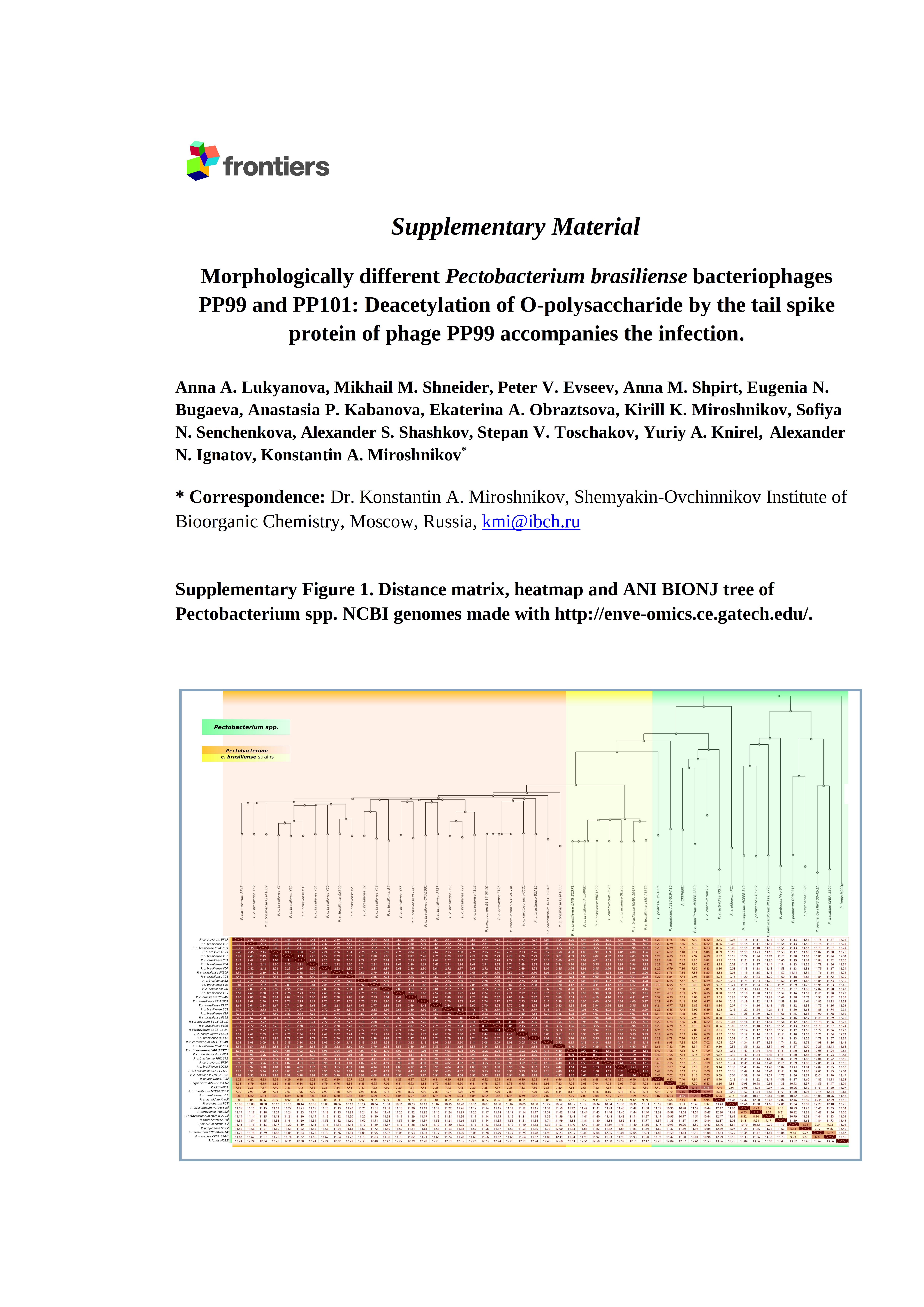

Supplement: Supplementary file 1 [file Image_1.JPEG]

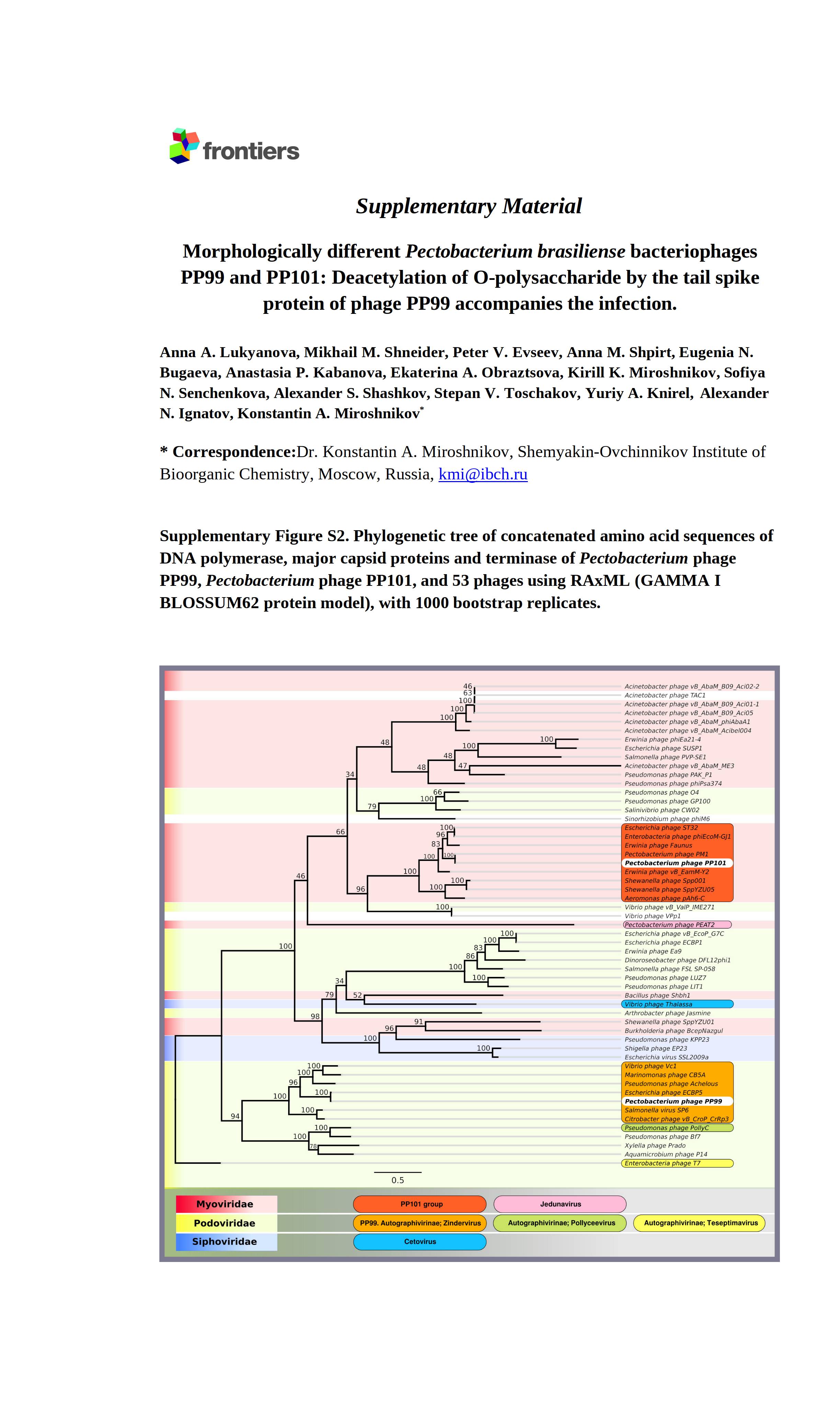

Supplement: Supplementary file 2 [file Image_2.JPEG]

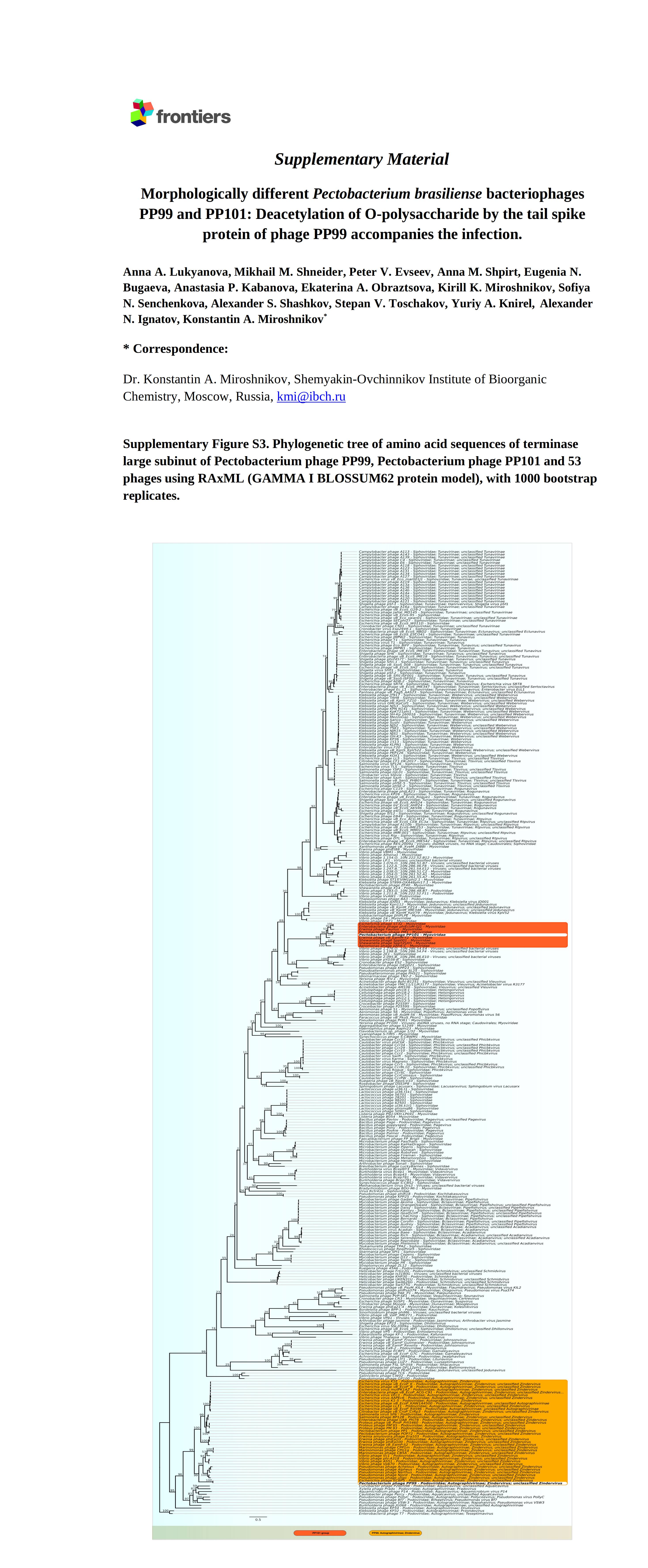

Supplement: Supplementary file 3 [file Image_3.JPEG]

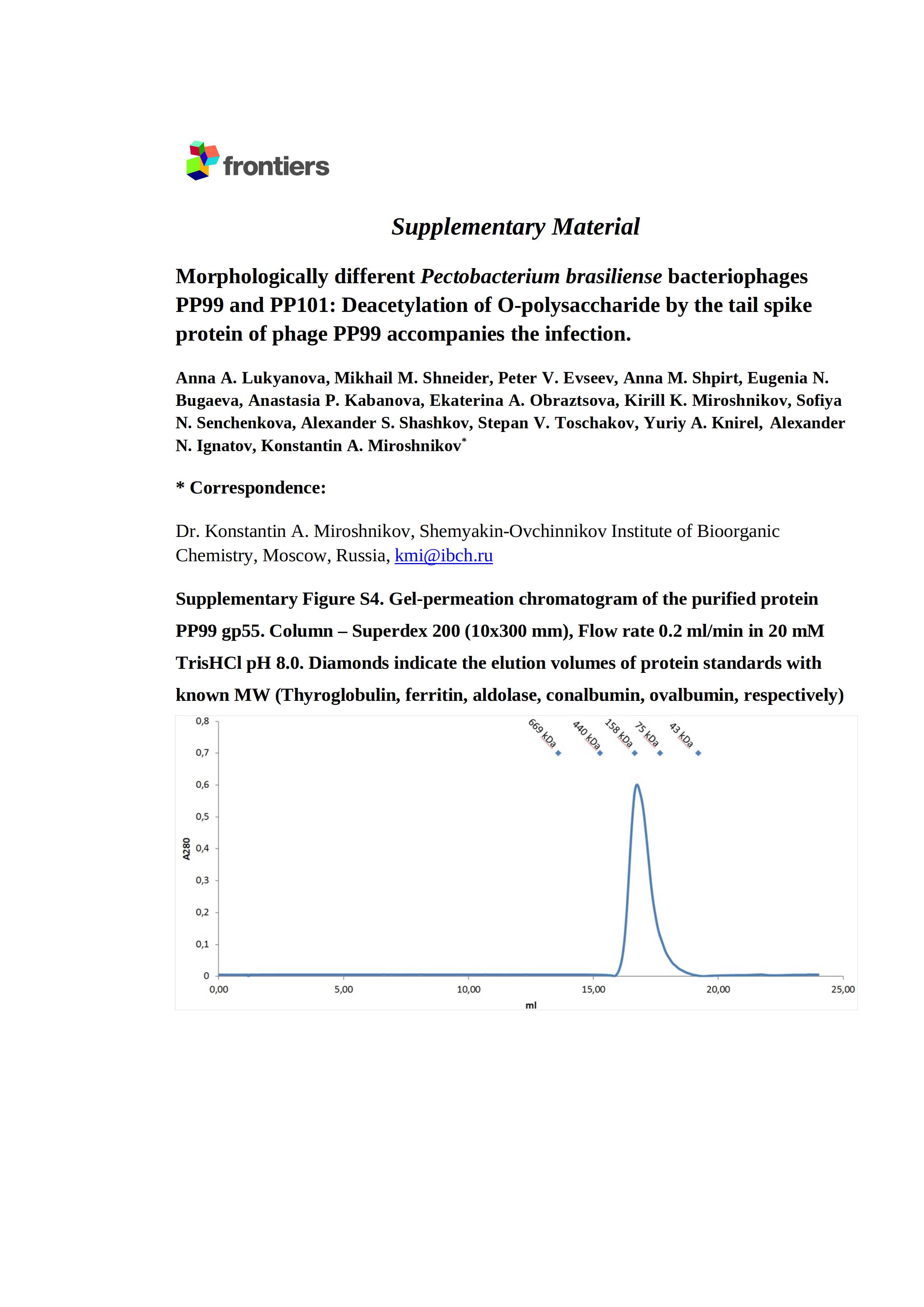

Supplement: Supplementary file 4 [file Image_4.JPEG]
