# Supplementary material for: Morphologically Different Pectobacterium brasiliense Bacteriophages PP99 and PP101: Deacetylation of O-Polysaccharide by the Tail Spike Protein of Phage PP99 Accompanies the Infection
Source: Front Microbiol. 2020 Jan 23;10:3147. doi: 10.3389/fmicb.2019.03147 (PMC6989608; doi:10.3389/fmicb.2019.03147)
Supplement: Supplementary file 5 [file Table_1.DOCX]

Supplementary Material

Morphologically different *Pectobacterium brasiliense* bacteriophages PP99 and PP101: Deacetylation of O-polysaccharide by the tail spike protein of phage PP99 accompanies the infection.

Anna A. Lukyanova, Mikhail M. Shneide, Peter V. Evseev, Anna M. Shpirt, Eugenia N. Bugaeva, Anastasia P. Kabanova, Ekaterina A. Obraztsova, Kirill K. Miroshnikov, Sofiya N. Senchenkova, Alexander S. Shashkov, Stepan V. Toschakov, Yuriy A. Knirel, Alexander N. Ignatov, Konstantin A. Miroshnikov^*^

*** Correspondence:**

Dr. Konstantin A. Miroshnikov, Shemyakin-Ovchinnikov Institute of Bioorganic Chemistry, Moscow, Russia, [kmi@ibch.ru](mailto:kmi@ibch.ru)

**Supplementary Table 1.** Infection range of bacteriophages PP99 and PP101

| # | Strain designation | Species | Year of isolation | Geographical region | Genus/species determination | PP99 | PP101 |
| --- | --- | --- | --- | --- | --- | --- | --- |
| 1 | F152 (PB29) | Pbr | 2014 | Moscow | NZ_PJDM00000000.1 | + | + |
| 2 | F126 | Pbr | 2012 | Samara | NZ_RRYQ00000000.1 | + | + |
| 3 | F128 | Pbr | 2012 | Samara | 16S, PCR | + | + |
| 4 | F157 (PB38) | Pbr | 2015 | Moscow | NZ_PJDL00000000.1 | + | + |
| 5 | F002  (PB69) | Pve | 2012 | Moscow | NZ_PDVY00000000.1 | + | + |
| 6 | F018 | Pve | 1947 | Moscow | NZ_PDVV00000000.1 | + | + |
| 7 | F022 | Pve | 2003 | Moscow | 16S, PCR | + | + |
| 8 | F040 | Pve | 2005 | Moscow | 16S, PCR | + | + |
| 9 | F058 | Pve | 2003 | Moscow | 16S, PCR | + | + |
| 10 | F072 | Pve | 2011 | Moscow | 16S, PCR | + | + |
| 11 | F100 | Pve | 1993 | Tver | 16S, PCR | - | + |
| 12 | F135 | Pve | 1995 | Vladimir | NZ_PDVX00000000.1 | + | + |
| 13 | F138 | Pve | 2005 | Moscow | 16S, PCR | + | + |
| 14 | F172 | Pve | 2018 | N. Novgorod | 16S, PCR | + | + |
| 15 | F012 | Dso | 2010 | Voronezh | NZ_PGOJ00000.1 | - | - |
| 16 | F043 | Dso | 2010 | Moscow | 16S , PCR | - | - |
| 17 | F156 | Dso | 2014 | Moscow | 16S, PCR | - | - |
| 18 | D12 | Dso | 2012 | Moscow | NZ_PGUT00000.1 | - | - |
| 29 | F069 | Ddi | 2011 | Kashira | 16S, PCR | - | - |
| 20 | F077 | Ddi | 2002 | Tyumen | 16S, PCR | - | - |
| 21 | F085 | Ddi | 1979 | Moscow | NZ_RSBK00000000.1 | - | - |
| 22 | F090 | Ddi | 1995 | Kaluga | 16S, PCR | - | - |
| 23 | SCRI1043 | Pat | ND | Scotland | NC_004547 | - | - |
| 24 | 21A | Pat | 1990 | Belarus | NZ_CP009125 | - | - |
| 25 | F004 (PB72) | Pat | 2012 | Moscow | NZ_PDDK00000000.1 | - | - |
| 26 | F035 | Ppa | 2012 | Kaluga | 16S, PCR | - | - |
| 27 | F148 (PB20) | Ppa | 2013 | Moscow | NZ_PDDJ00000000.1 | - | - |
| 28 | F109 | Ppo | 1979 | Samara | NZ_RRYS00000000.1 | - | - |
| 29 | F171 | Ppo | 2018 | Moscow | 16S, PCR | - | - |
| 30 | F008 | Pcc | 1923 | VKPM | 16S, PCR | - | - |
| 31 | F160 (NCPPB312) | Pcc | ND | Moscow | NZ_JQHJ00000000.1 | - | - |
| 32 | F053 | Pcc | 2011 | Moscow | 16S, PCR | - | - |
| 33 | F118 | Pcc | 2005 | Moscow | 16S, PCR | - | - |
| 34 | F140 | Pcc | 2012 | Moscow | 16S, PCR | - | - |
| 35 | F150 | Pcc | 2014 | Moscow | 16S, PCR | - | - |
| 36 | F016 | Pve | 1993 | Ryazan | NZ_RRYR00000000.1 | - | - |
| 37 | F131 | Pve | 1998 | Kaluga | NZ_PDVW00000000.1 | - | - |
| 38 | F134 | Pve | 2002 | Tyumen | 16S, PCR | - | - |
| 39 | F164 (PB31) | Paq | 2016 | Moscow | NZ_PJJA00000000.1 | - | - |
| 40 | F096 | Pseudomonas | 1989 | Moscow | 16S | - | - |
| 41 | F039 | Pseudomonas | 1972 | Lithuania | 16S | - | - |
| 42 | F153 | Lelliottia | 2014 | Moscow | NZ_PKFT00000000.1 | - | - |
| 43 | F154 | Lelliottia | 2014 | Moscow | NZ_PKFV00000000.1 | - | - |
| 44 | F159 | Lelliottia | 2014 | Moscow | NZ_PKFU00000000.1 | - | - |
| 45 | F084 | Xanthomonas | 2005 | Moscow | 16S | - | - |
| 46 | F142 | Pantoea | 2013 | Moscow | 16S | - | - |

+ Denotes plaque formation on the respective host

Pve- The strain belongs to the new species *Pectobacterium versatile* (Portier et al., 2019) initially suggested as *Pectobacterium maceratum* (Shirshikov et al., 2018), previously regarded as a strain group of *Pectobacterium carortovorum* subsp*. carotovorum*

PCR methods used to verify the taxonomy of the isolates were the following: *Pectobacterium carortovorum* subsp*. carotovorum* (Pcc) - (Kang et al., 2003), *Pectobacterium atrosepticum* (Pat) - (De Boer and Ward, 1995), *Pectobacterium brasiliense* (Pbr) - (Duarte et al., 2004), *Pectobacterium parmentieri* (former *P.wasabiae*) - (De Boer et al., 2012), *Dickeya solani* (Dso) – conventional PCR adaptation from (van Vaerenbergh et al., 2012), *Dickeya dianthicola* (Ddi) - conventional PCR adaptation from (Pritchard et al., 2013)

**References**

De Boer, S. H., Li, X., and Ward, L. J. (2012). Pectobacterium spp. Associated with Bacterial Stem Rot Syndrome of Potato in Canada. *Phytopathology* 102, 937–947. doi:10.1094/PHYTO-04-12-0083-R.

De Boer, S. H., and Ward, L. J. (1995). PCR detection of Erwinia carotovora subsp atroseptica associated with potato tissue. *Phytopathology* 85, 854–858. doi:10.1094/Phyto-85-854.

Duarte, V., De Boer, S. H., Ward, L. J., and De Oliveira, A. M. R. (2004). Characterization of atypical Erwinia carotovora strains causing blackleg of potato in Brazil. *Journal of Applied Microbiology* 96, 535–545. doi:10.1111/j.1365-2672.2004.02173.x.

Kang, H. W., Kwon, S. W., and Go, S. J. (2003). PCR-based specific and sensitive detection of Pectobacterium carotovorum ssp. carotovorum by primers generated from a URP-PCR fingerprinting-derived polymorphic band. *Plant Pathology* 52, 127–133. doi:10.1046/j.1365-3059.2003.00822.x.

Portier, P., Pédron, J., Taghouti, G., Fischer-Le Saux, M., Caullireau, E., Bertrand, C., et al. (2019). Elevation of Pectobacterium carotovorum subsp. odoriferum to species level as Pectobacterium odoriferum sp. nov., proposal of Pectobacterium brasiliense sp. nov. and Pectobacterium actinidiae sp. nov., emended description of Pectobacterium carotovorum and description of Pectobacterium versatile sp. nov., isolated from streams and symptoms on diverse plants. *International journal of systematic and evolutionary microbiology*. doi:10.1099/ijsem.0.003611.

Pritchard, L., Humphris, S., Saddler, G. S., Parkinson, N. M., Bertrand, V., Elphinstone, J. G., et al. (2013). Detection of phytopathogens of the genus Dickeya using a PCR primer prediction pipeline for draft bacterial genome sequences. *Plant Pathology* 62, 587–596. doi:10.1111/j.1365-3059.2012.02678.x.

Shirshikov, F. V., Korzhenkov, A. A., Miroshnikov, K. K., Kabanova, A. P., Barannik, A. P., Ignatov, A. N., et al. (2018). Draft Genome Sequences of New Genomospecies “ *Candidatus* Pectobacterium maceratum” Strains, Which Cause Soft Rot in Plants. *Genome Announcements* 6, e00260-18. doi:10.1128/genomeA.00260-18.

van Vaerenbergh, J., Baeyen, S., de Vos, P., and Maes, M. (2012). Sequence diversity in the Dickeya flic gene: Phylogeny of the Dickeya genus and taqman® PCR for “D. solani”, new biovar 3 variant on potato in Europe. *PLoS ONE* 7. doi:10.1371/journal.pone.0035738.
