# Supplementary material for: Morphologically Different Pectobacterium brasiliense Bacteriophages PP99 and PP101: Deacetylation of O-Polysaccharide by the Tail Spike Protein of Phage PP99 Accompanies the Infection
Source: Front Microbiol. 2020 Jan 23;10:3147. doi: 10.3389/fmicb.2019.03147 (PMC6989608; doi:10.3389/fmicb.2019.03147)
Supplement: Supplementary file 6 [file Table_2.DOCX]

Supplementary Material

Morphologically different *Pectobacterium brasiliense* bacteriophages PP99 and PP101: Deacetylation of O-polysaccharide by the tail spike protein of phage PP99 accompanies the infection.

Anna A. Lukyanova, Mikhail M. Shneider, Peter V. Evseev, Anna M. Shpirt, Eugenia N. Bugaeva, Anastasia P. Kabanova, Ekaterina A. Obraztsova, Kirill K. Miroshnikov, Sofiya N. Senchenkova, Alexander S. Shashkov, Stepan V. Toschakov, Yuriy A. Knirel, Alexander N. Ignatov, Konstantin A. Miroshnikov

**Supplementary Table 2.** Putative gene functions of *Pectobacterium brasiliense* bacteriophage PP99 (vB_PbrP_PP99), 44609 bp genome, 56 ORFs.

| **locus_tag** | **Start** | **End** | **Translation, aa** | **Predicted function** | **Closest relative** | **Query Cover** | **E value** | **% Ident** |
| --- | --- | --- | --- | --- | --- | --- | --- | --- |
| PP99_01 | 790 | 1035 | 82 | hypothetical protein | penicillin-binding protein [Candidatus Stoquefichus massiliensis] | 64% | 0,37 | 36,54% |
| PP99_02 | 1179 | 1361 | 61 | hypothetical protein | hypothetical protein GA2A_01 [Escherichia phage vB_EcoP_GA2A] | 96% | 3,00E-20 | 63,79% |
| PP99_03 | 1679 | 2020 | 114 | hypothetical protein | hypothetical protein [Dysgonomonas macrotermitis] | 94% | 9,00E-05 | 31,48% |
| PP99_04 | 2087 | 2242 | 52 | hypothetical protein | - | - | - | - |
| PP99_05 | 2287 | 2475 | 63 | hypothetical protein | hypothetical protein ECBP5_0062 [Escherichia phage ECBP5] | 100% | 3,00E-37 | 98,39% |
| PP99_06 | 2477 | 2707 | 77 | hypothetical protein | hypothetical protein ECBP5_0001 [Escherichia phage ECBP5] | 69% | 0,02 | 37,50% |
| PP99_07 | 2835 | 2999 | 55 | hypothetical protein | - | - | - | - |
| PP99_08 | 3198 | 4232 | 345 | hypothetical protein | hypothetical protein ECBP5_0004 [Escherichia phage ECBP5] | 100% | 0 | 93,90% |
| PP99_09 | 4229 | 4405 | 59 | hypothetical protein | hypothetical protein DRP82_03165 [Planctomycetes bacterium] | 46% | 1,8 | 40,74% |
| PP99_10 | 4455 | 7088 | 878 | DNA-directed RNA polymerase | putative RNA polymerase [Escherichia phage ECBP5] | 100% | 0 | 99,20% |
| PP99_11 | 7098 | 7502 | 135 | hypothetical protein | hypothetical protein ECBP5_0007 [Escherichia phage ECBP5] | 99% | 6,00E-80 | 86,47% |
| PP99_12 | 7502 | 7678 | 59 | hypothetical protein | hypothetical protein ECBP5_0008 [Escherichia phage ECBP5] | 100% | 3,00E-27 | 81,03% |
| PP99_13 | 7675 | 7887 | 71 | hypothetical protein | hypothetical protein ECBP5_0010 [Escherichia phage ECBP5] | 97% | 2,00E-38 | 94,12% |
| PP99_14 | 8332 | 8496 | 55 | hypothetical protein | hypothetical protein ECBP5_0012 [Escherichia phage ECBP5] | 92% | 1,00E-25 | 98,00% |
| PP99_15 | 8496 | 8672 | 59 | hypothetical protein | Aspartate decarboxylase-like domain-containing protein [Cynara cardunculus var. scolymus] | 55% | 2,1 | 42,50% |
| PP99_16 | 8672 | 10684 | 671 | putative DNA primase/helicase | putative DNA helicase [Escherichia phage ECBP5] | 96% | 0 | 98,30% |
| PP99_17 | 10684 | 11154 | 157 | putative ssDNA binding protein | DUF1273 family protein [Massilia alkalitolerans] | 98% | 7,00E-49 | 48,05% |
| PP99_18 | 11147 | 11410 | 88 | hypothetical protein | hypothetical protein ECBP5_0015 [Escherichia phage ECBP5] | 100% | 3,00E-31 | 71,26% |
| PP99_19 | 11410 | 11607 | 66 | hypothetical protein | hypothetical protein ECBP5_0018 [Escherichia phage ECBP5] | 100% | 1,00E-37 | 98,46% |
| PP99_20 | 11653 | 11925 | 91 | hypothetical protein | hypothetical protein ECBP5_0020 [Escherichia phage ECBP5] | 100% | 2,00E-52 | 88,89% |
| PP99_21 | 11918 | 12571 | 218 | putative nucleotidyltransferase | hypothetical protein ECBP5_0021 [Escherichia phage ECBP5] | 96% | 6,00E-111 | 73,21% |
| PP99_22 | 12599 | 15121 | 841 | DNA polymerase | putative DNA polymerase [Escherichia phage ECBP5] | 100% | 0 | 91,23% |
| PP99_23 | 15170 | 15424 | 85 | hypothetical protein | hypothetical protein ECBP5_0023 [Escherichia phage ECBP5] | 86% | 8,00E-13 | 45,21% |
| PP99_24 | 15512 | 16276 | 255 | hypothetical protein | hypothetical protein ECBP5_0024 [Escherichia phage ECBP5] | 100% | 3,00E-154 | 83,46% |
| PP99_25 | 16488 | 16838 | 117 | hypothetical protein | hypothetical protein ECBP5_0025 [Escherichia phage ECBP5] | 88% | 7,00E-60 | 91,26% |
| PP99_26 | 16893 | 17981 | 363 | 5'-3'exonuclease | putative exonuclease [Escherichia phage ECBP5] | 100% | 0 | 90,06% |
| PP99_27 | 17972 | 18379 | 136 | endonuclease VII | putative endonuclease [Escherichia phage ECBP5] | 100% | 1,00E-82 | 87,41% |
| PP99_28 | 18382 | 19503 | 374 | putative exonuclease | putative oxidoreductase [Escherichia phage ECBP5] | 100% | 0 | 94,10% |
| PP99_29 | 19543 | 20121 | 193 | putative phosphatase | putative polynucleotide 5' kinase/3' phosphatase [Escherichia phage ECBP5] | 100% | 7,00E-124 | 88,21% |
| PP99_30 | 20118 | 20699 | 194 | putative deoxynucleoside monophosphate kinase | hypothetical protein ECBP5_0030 [Escherichia phage ECBP5] | 97% | 2,00E-131 | 95,74% |
| PP99_31 | 20686 | 21639 | 318 | DNA ligase | hypothetical protein ECBP5_0031 [Escherichia phage ECBP5] | 100% | 0 | 94,32% |
| PP99_32 | 21614 | 22021 | 136 | hypothetical protein | - | - | - | - |
| PP99_33 | 22005 | 22181 | 59 | hypothetical protein | hypothetical protein ECBP5_0033 [Escherichia phage ECBP5] | 100% | 2,00E-30 | 94,83% |
| PP99_34 | 22135 | 22365 | 77 | hypothetical protein | hypothetical protein ECBP5_0034 [Escherichia phage ECBP5] | 100% | 8,00E-45 | 92,11% |
| PP99_35 | 22344 | 22805 | 154 | putative acyl-CoA N-acyltransferase domain-containing protein | putative acyl-CoA N-acyltransferase domain-containing protein [Escherichia phage ECBP5] | 100% | 3,00E-109 | 98,69% |
| PP99_36 | 22802 | 23008 | 69 | hypothetical protein | hypothetical protein ECBP5_0036 [Escherichia phage ECBP5] | 100% | 4,00E-39 | 98,53% |
| PP99_37 | 23018 | 24610 | 531 | putative head-tail connector protein | putative head-tail connector [Escherichia phage ECBP5] | 100% | 0 | 97,55% |
| PP99_38 | 24621 | 25499 | 293 | putative scaffolding protein | putative scaffolding protein [Escherichia phage ECBP5] | 100% | 0 | 96,92% |
| PP99_39 | 25600 | 26712 | 371 | putative major capsid protein | putative major capsid protein [Escherichia phage ECBP5] | 100% | 0 | 99,46% |
| PP99_40 | 26772 | 27401 | 210 | putative tail tubular protein A | putative tail tubular protein A [Escherichia phage ECBP5] | 100% | 4,00E-153 | 98,56% |
| PP99_41 | 27410 | 29800 | 797 | putative tail tubular protein B | putative tail tubular protein B [Escherichia phage ECBP5] | 100% | 0 | 97,99% |
| PP99_42 | 29766 | 30416 | 217 | putative internal virion protein A | hypothetical protein ECBP5_0042 [Escherichia phage ECBP5] | 77% | 1,00E-97 | 94,64% |
| PP99_43 | 30416 | 32560 | 715 | putative internal virion protein B | hypothetical protein ECBP5_0043 [Escherichia phage ECBP5] | 98% | 0 | 96,45% |
| PP99_44 | 32570 | 36436 | 1289 | putative internal virion protein C | putative internal virion-like protein [Escherichia phage ECBP5] | 100% | 0 | 90,85% |
| PP99_45 | 36444 | 37364 | 307 | tail fiber adaptor protein | putative tail fiber protein [Escherichia phage ECBP5] | 100% | 0 | 97,71% |
| PP99_46 | 37734 | 37913 | 60 | putative small terminase subunit-like protein | putative small terminase subunit-like protein [Escherichia phage ECBP5] | 100% | 4,00E-32 | 93,22% |
| PP99_47 | 37910 | 39760 | 617 | terminase large subunit | putative large terminase subunit-like protein [Escherichia phage ECBP5] | 100% | 0 | 99,51% |
| PP99_48 | 39934 | 40545 | 204 | tail needle and knob protein | hypothetical protein ECBP5_0049 [Escherichia phage ECBP5] | 100% | 6,00E-134 | 95,07% |
| PP99_49 | 40559 | 40837 | 93 | hypothetical protein | putativeacetyl-CoA acetyltransferase [Escherichia phage ECBP5] | 100% | 2,00E-52 | 90,22% |
| PP99_50 | 40881 | 41438 | 186 | lytic murein transglycosylase | hypothetical protein ECBP5_0051 [Escherichia phage ECBP5] | 100% | 5,00E-132 | 96,22% |
| PP99_51 | 41551 | 41865 | 105 | hypothetical protein | hypothetical protein ECBP5_0053 [Escherichia phage ECBP5] | 100% | 1,00E-59 | 88,46% |
| PP99_52 | 41874 | 42023 | 50 | hypothetical protein | hypothetical protein ECBP5_0054 [Escherichia phage ECBP5] | 100% | 1,00E-21 | 91,84% |
| PP99_53 | 42023 | 42232 | 70 | hypothetical protein | membrane protein [Proteus phage PM 93] | 89% | 2,00E-05 | 38,71% |
| PP99_54 | 42102 | 42311 | 70 | hypothetical protein | hypothetical protein ECBP5_0055 [Escherichia phage ECBP5] | 100% | 3,00E-35 | 88,41% |
| PP99_55 | 42368 | 44020 | 551 | tail spike protein | SGNH/GDSL hydrolase family protein [Enterobacter cloacae] | 88% | 1,00E-66 | 33,67% |
| PP99_56 | 44122 | 44352 | 77 | hypothetical protein | DUF2829 domain-containing protein [Acinetobacter sp. RIT592] | 90% | 6,00E-11 | 40,28% |
