# Supplementary material for: Morphologically Different Pectobacterium brasiliense Bacteriophages PP99 and PP101: Deacetylation of O-Polysaccharide by the Tail Spike Protein of Phage PP99 Accompanies the Infection
Source: Front Microbiol. 2020 Jan 23;10:3147. doi: 10.3389/fmicb.2019.03147 (PMC6989608; doi:10.3389/fmicb.2019.03147)
Supplement: Supplementary file 7 [file Table_3.DOCX]

Supplementary Material

Morphologically different *Pectobacterium brasiliense* bacteriophages PP99 and PP101: Deacetylation of O-polysaccharide by the tail spike protein of phage PP99 accompanies the infection.

Anna A. Lukyanova, Mikhail M. Shneider, Peter V. Evseev, Anna M. Shpirt, Eugenia N. Bugaeva, Anastasia P. Kabanova, Ekaterina A. Obraztsova, Kirill K. Miroshnikov, Sofiya N. Senchenkova, Alexander S. Shashkov, Stepan V. Toschakov, Yuriy A. Knirel, Alexander N. Ignatov, Konstantin A. Miroshnikov

**Supplementary Table 3.** Putative gene functions of *Pectobacterium brasiliense* bacteriophages PP101 (vB_PbrM_PP101), 53333 bp genome, 82 ORFs.

| **locus_tag** | **Start** | **End** | **Translation, aa** | **Predicted function** | **Closest relative** | **Query Cover** | **E value** | **% Ident** |
| --- | --- | --- | --- | --- | --- | --- | --- | --- |
| PP101_01 | 396 | 2321 | 642 | DNA-dependent RNA polymerase | DNA-dependent RNA polymerase [Pectobacterium phage PM1] | 100% | 0 | 99,38% |
| PP101_02 | 3606 | 3950 | 115 | hypothetical protein | hypothetical protein PM1_002 [Pectobacterium phage PM1] | 100% | 3,00E-65 | 89,66% |
| PP101_03 | 3947 | 4135 | 63 | hypothetical protein | hypothetical protein [Erwinia phage Faunus] | 98% | 3,00E-16 | 50,82% |
| PP101_04 | 4132 | 4383 | 84 | hypothetical protein | hypothetical protein M404DRAFT_1005441 [Pisolithus tinctorius Marx 270] | 65% | 1,1 | 37,04% |
| PP101_05 | 4370 | 4528 | 53 | hypothetical protein | methyl-accepting chemotaxis protein [Roseburia intestinalis] | 63% | 5,3 | 48,48% |
| PP101_06 | 4525 | 4743 | 73 | hypothetical protein | hypothetical protein PM1_003 [Pectobacterium phage PM1] | 100% | 8,00E-40 | 93,06% |
| PP101_07 | 4730 | 4906 | 59 | hypothetical protein | - | - | - | - |
| PP101_08 | 5065 | 5313 | 83 | hypothetical protein | hypothetical protein PM1_004 [Pectobacterium phage PM1] | 100% | 1,00E-49 | 95,12% |
| PP101_09 | 5310 | 5588 | 93 | hypothetical protein | hypothetical protein PM1_005 [Pectobacterium phage PM1] | 100% | 5,00E-56 | 88,04% |
| PP101_10 | 5585 | 5806 | 74 | hypothetical protein | AAA family ATPase [Bacillus flexus] | 67% | 0,83 | 44,00% |
| PP101_11 | 5897 | 6079 | 61 | hypothetical protein | hypothetical protein DYB32_007020 [Aphanomyces invadans] | 81% | 3,5 | 32,69% |
| PP101_12 | 6076 | 6363 | 96 | hypothetical protein | hypothetical protein PM1_006 [Pectobacterium phage PM1] | 100% | 1,00E-62 | 97,89% |
| PP101_13 | 6750 | 7061 | 104 | hypothetical protein | NrdA.1-like protein [Cronobacter phage CR9] | 98% | 5,00E-27 | 51,46% |
| PP101_14 | 7183 | 7557 | 125 | putative single-stranded DNA binding protein | putative single-stranded DNA-binding protein [Pectobacterium phage PM1] | 100% | 2,00E-81 | 96,77% |
| PP101_15 | 7557 | 7985 | 143 | hypothetical protein | hypothetical protein PM1_009 [Pectobacterium phage PM1] | 100% | 2,00E-98 | 98,59% |
| PP101_16 | 8049 | 8510 | 154 | hypothetical protein | hypothetical protein EPR50_G00058350 [Perca flavescens] | 79% | 3,2 | 28,23% |
| PP101_17 | 8507 | 8917 | 137 | hypothetical protein | hypothetical protein PM1_010 [Pectobacterium phage PM1] | 94% | 2,00E-65 | 73,64% |
| PP101_18 | 8902 | 9117 | 72 | hypothetical protein | - | - | - | - |
| PP101_19 | 9155 | 9460 | 102 | hypothetical protein | hypothetical protein PM1_011 [Pectobacterium phage PM1] | 98% | 3,00E-48 | 76,77% |
| PP101_20 | 9460 | 9738 | 93 | hypothetical protein | - | - | - | - |
| PP101_21 | 9739 | 10215 | 159 | putative peptidoglycan hydrolase | TIGR02594 family protein [Pectobacterium phage PM1] | 100% | 1,00E-109 | 96,84% |
| PP101_22 | 10215 | 10526 | 104 | hypothetical protein | GGDEF domain-containing protein [Merismopedia glauca] | 45% | 0,17 | 36,17% |
| PP101_23 | 10546 | 10830 | 95 | hypothetical protein | sensor histidine kinase [Vibrio caribbeanicus] | 57% | 2 | 36,36% |
| PP101_24 | 10873 | 11439 | 189 | putative antirestriction protein | putative antirestriction protein [Pectobacterium phage PM1] | 100% | 1,00E-133 | 98,40% |
| PP101_25 | 11423 | 11680 | 86 | hypothetical protein | hypothetical protein [Erwinia phage Faunus] | 88% | 1,00E-12 | 38,96% |
| PP101_26 | 11776 | 11907 | 44 | hypothetical protein | - | - | - | - |
| PP101_27 | 12620 | 12970 | 117 | hypothetical protein | hypothetical protein PM1_015 [Pectobacterium phage PM1] | 100% | 6,00E-77 | 95,69% |
| PP101_28 | 12967 | 13227 | 87 | hypothetical protein | olfactory receptor 5A1-like [Gopherus evgoodei] | 56% | 5,5 | 34,55% |
| PP101_29 | 13227 | 13532 | 102 | hypothetical protein | hypothetical protein PM1_017 [Pectobacterium phage PM1] | 99% | 3,00E-63 | 97,00% |
| PP101_30 | 13534 | 14205 | 224 | hypothetical protein | hypothetical protein PM1_018 [Pectobacterium phage PM1] | 100% | 6,00E-163 | 99,55% |
| PP101_31 | 14327 | 14686 | 120 | hypothetical protein | hypothetical protein PM1_019 [Pectobacterium phage PM1] | 100% | 1,00E-76 | 97,48% |
| PP101_32 | 15466 | 15678 | 71 | hypothetical protein | hypothetical protein DRH37_03115 [Deltaproteobacteria bacterium] | 81% | 0,25 | 36,84% |
| PP101_33 | 15662 | 15934 | 91 | hypothetical protein | hypothetical protein PM1_021 [Pectobacterium phage PM1] | 100% | 4,00E-44 | 94,44% |
| PP101_34 | 15795 | 16070 | 92 | hypothetical protein | hypothetical protein PM1_022 [Pectobacterium phage PM1] | 64% | 2,00E-35 | 94,92% |
| PP101_35 | 16031 | 16147 | 39 | hypothetical protein | - | - | - | - |
| PP101_36 | 16645 | 16806 | 54 | hypothetical protein | - | - | - | - |
| PP101_37 | 17108 | 17383 | 92 | hypothetical protein | hypothetical protein PM1_023 [Pectobacterium phage PM1] | 74% | 4,00E-19 | 62,86% |
| PP101_38 | 17432 | 18049 | 206 | putative thymidylate synthase | thymidylate synthase [Pectobacterium phage PM1] | 100% | 3,00E-133 | 89,76% |
| PP101_39 | 18063 | 19823 | 587 | putative DNA primase/helicase | DNA primase/helicase [Pectobacterium phage PM1] | 95% | 0 | 99,29% |
| PP101_40 | 19893 | 21827 | 645 | putative DNA polymerase | DNA polymerase [Pectobacterium phage PM1] | 100% | 0 | 97,98% |
| PP101_41 | 21827 | 22075 | 83 | hypothetical protein | hypothetical protein BSQ96_10375 [Serratia proteamaculans] | 39% | 4,6 | 48,48% |
| PP101_42 | 22094 | 22948 | 285 | hypothetical protein | hypothetical protein PM1_027 [Pectobacterium phage PM1] | 100% | 0 | 99,65% |
| PP101_43 | 23094 | 24074 | 327 | putative 5'-3' exonuclease | 5'-3' exonuclease [Pectobacterium phage PM1] | 100% | 0 | 98,77% |
| PP101_44 | 24119 | 24643 | 175 | putative endonuclease VII | HNH domain containing protein [Pectobacterium phage PM1] | 100% | 3,00E-124 | 98,85% |
| PP101_45 | 24633 | 25400 | 256 | DNA ligase | putative DNA ligase [Pectobacterium phage PM1] | 100% | 0 | 96,47% |
| PP101_46 | 25400 | 25612 | 71 | hypothetical protein | glycosyltransferase family 4 protein [Paenarthrobacter nicotinovorans] | 82% | 3,4 | 38,33% |
| PP101_47 | 25609 | 26199 | 197 | putative deoxynucleoside monophosphate kinase | putative ATP-binding protein [Pectobacterium phage PM1] | 100% | 2,00E-134 | 95,41% |
| PP101_48 | 26199 | 26648 | 150 | putative deoxyuridine 5'-triphosphate nucleotidohydrolase | deoxyuridine 5'-triphosphate nucleotidohydrolase [Pectobacterium phage PM1] | 100% | 1,00E-102 | 94,63% |
| PP101_49 | 26667 | 26855 | 63 | putative host recBCD nuclease inhibitor | hypothetical protein PM1_033 [Pectobacterium phage PM1] | 67% | 4,00E-19 | 95,24% |
| PP101_50 | 26848 | 27243 | 132 | hypothetical protein | hypothetical protein PM1_034 [Pectobacterium phage PM1] | 100% | 1,00E-90 | 98,47% |
| PP101_51 | 27317 | 27541 | 75 | hypothetical protein | hypothetical protein PM1_035 [Pectobacterium phage PM1] | 100% | 2,00E-45 | 97,30% |
| PP101_52 | 27604 | 27786 | 61 | hypothetical protein | hypothetical protein PM1_036 [Pectobacterium phage PM1] | 100% | 1,00E-35 | 96,67% |
| PP101_53 | 27796 | 29808 | 671 | putative terminase large subunit | terminase large subunit [Pectobacterium phage PM1] | 100% | 0 | 99,25% |
| PP101_54 | 29810 | 30025 | 72 | hypothetical protein | hypothetical protein PM1_038 [Pectobacterium phage PM1] | 100% | 6,00E-43 | 100,00% |
| PP101_56 | 30026 | 31360 | 445 | putative portal protein | portal protein [Pectobacterium phage PM1] | 100% | 0 | 99,32% |
| PP101_57 | 31323 | 32387 | 355 | putative prohead protease | putative head protein [Pectobacterium phage PM1] | 99% | 0 | 96,59% |
| PP101_58 | 31386 | 32387 | 334 | hypothetical protein | putative head protein [Pectobacterium phage PM1] | 100% | 0 | 96,70% |
| PP101_59 | 32399 | 32872 | 158 | hypothetical protein | hypothetical protein PM1_041 [Pectobacterium phage PM1] | 100% | 4,00E-106 | 97,45% |
| PP101_60 | 33203 | 34216 | 338 | putative major capsid protein | major capsid protein [Pectobacterium phage PM1] | 100% | 0 | 97,92% |
| PP101_61 | 34269 | 34706 | 146 | hypothetical protein | hypothetical protein PM1_043 [Pectobacterium phage PM1] | 100% | 3,00E-89 | 90,34% |
| PP101_62 | 34706 | 35083 | 126 | putative head-tail connector protein | gp70 [Erwinia phage vB_EamM-Y2] | 100% | 2,00E-54 | 67,20% |
| PP101_63 | 35092 | 35454 | 121 | putative head-tail adaptor protein | hypothetical protein PM1_045 [Pectobacterium phage PM1] | 100% | 4,00E-84 | 99,17% |
| PP101_64 | 35455 | 35970 | 172 | putative tail completion protein | hypothetical protein PM1_046 [Pectobacterium phage PM1] | 100% | 4,00E-122 | 97,66% |
| PP101_65 | 35971 | 37419 | 483 | tail sheath protein | hypothetical protein PM1_047 [Pectobacterium phage PM1] | 100% | 0 | 97,10% |
| PP101_66 | 37429 | 37884 | 152 | tail tube protein | hypothetical protein PM1_048 [Pectobacterium phage PM1] | 100% | 6,00E-106 | 100,00% |
| PP101_67 | 37900 | 38352 | 151 | putative tail protein | hypothetical protein PM1_049 [Pectobacterium phage PM1] | 100% | 2,00E-104 | 100,00% |
| PP101_68 | 38370 | 38528 | 53 | hypothetical protein | hypothetical protein PM1_050 [Pectobacterium phage PM1] | 100% | 5,00E-30 | 100,00% |
| PP101_69 | 38518 | 42357 | 1280 | tape measure protein | putative tail fiber [Pectobacterium phage PM1] | 100% | 0 | 98,51% |
| PP101_70 | 42434 | 43531 | 366 | baseplate protein | hypothetical protein PM1_052 [Pectobacterium phage PM1] | 100% | 0 | 98,36% |
| PP101_71 | 43531 | 44430 | 300 | tail tube intiator | hypothetical protein PM1_053 [Pectobacterium phage PM1] | 100% | 0 | 99,00% |
| PP101_72 | 44427 | 44798 | 124 | putative minor baseplate protein | hypothetical protein PM1_054 [Pectobacterium phage PM1] | 100% | 8,00E-84 | 97,56% |
| PP101_73 | 44785 | 45570 | 262 | baseplate central spike | putative baseplate assembly protein [Pectobacterium phage PM1] | 100% | 0 | 98,08% |
| PP101_74 | 45570 | 45938 | 123 | tail sheath polimerization initiator | hypothetical protein PM1_056 [Pectobacterium phage PM1] | 100% | 1,00E-83 | 98,36% |
| PP101_75 | 45914 | 47080 | 389 | mail baseplate wedge protein | hypothetical protein PM1_057 [Pectobacterium phage PM1] | 100% | 0 | 97,94% |
| PP101_76 | 47088 | 47735 | 216 | baseplate protein | hypothetical protein PM1_058 [Pectobacterium phage PM1] | 100% | 7,00E-154 | 98,14% |
| PP101_77 | 47732 | 48886 | 385 | putative tail fiber protein | hypothetical protein PM1_059 [Pectobacterium phage PM1] | 100% | 0 | 98,70% |
| PP101_78 | 48886 | 50247 | 454 | putative tail fiber protein | hypothetical protein PM1_060 [Pectobacterium phage PM1] | 100% | 0 | 98,45% |
| PP101_79 | 50257 | 52137 | 627 | putative tail fiber protein | hypothetical protein PM1_061 [Pectobacterium phage PM1] | 100% | 0 | 60,00% |
| PP101_80 | 52148 | 52489 | 114 | putative holin | holin [Pectobacterium phage PM1] | 100% | 9,00E-76 | 100,00% |
| PP101_81 | 52507 | 53067 | 187 | lytic transglycosylase | putative endolysin [Pectobacterium phage PM1] | 99% | 8,00E-135 | 98,92% |
