# Supplementary material for: Morphologically Different Pectobacterium brasiliense Bacteriophages PP99 and PP101: Deacetylation of O-Polysaccharide by the Tail Spike Protein of Phage PP99 Accompanies the Infection
Source: Front Microbiol. 2020 Jan 23;10:3147. doi: 10.3389/fmicb.2019.03147 (PMC6989608; doi:10.3389/fmicb.2019.03147)
Supplement: Supplementary file 8 [file Table_4.DOCX]

Supplementary Material

Morphologically different *Pectobacterium brasiliense* bacteriophages PP99 and PP101: Deacetylation of O-polysaccharide by the tail spike protein of phage PP99 accompanies the infection.

Anna A. Lukyanova, Mikhail M. Shneide, Peter V. Evseev, Anna M. Shpirt, Eugenia N. Bugaeva, Anastasia P. Kabanova, Ekaterina A. Obraztsova, Kirill K. Miroshnikov, Sofiya N. Senchenkova, Alexander S. Shashkov, Stepan V. Toschakov, Yuriy A. Knirel, Alexander N. Ignatov, Konstantin A. Miroshnikov^*^

*** Correspondence:** Dr. Konstantin A. Miroshnikov, Shemyakin-Ovchinnikov Institute of Bioorganic Chemistry, Moscow, Russia, [kmi@ibch.ru](mailto:kmi@ibch.ru)

**Table S4.** ^1^H and ^13^C NMR chemical shifts (δ, ppm) of the O-deacetylated polysaccharide (DPS)

| Sugar residue | C-1  *H-1* | C-2  *H-2* | C-3  *H-3* | C-4  *H-4* | C-5  *H-5* | C-6  *H-6 (6a,6b)* |
| --- | --- | --- | --- | --- | --- | --- |
| →3)-β-d-Gal*p*-(1→  **A** | 103.7  *4.44* | 71.3  *3.53* | 77.7  *3.75* | 65.5  *4.12* | 76.2  *3.57* | 62.9  *3.73, 3.77* |
| →2)-α-d-Man*p*-(1→  **B** | 95.9  *5.27* | 80.4  *4.04* | 71.3  *4.04* | 68.1  *3.78* | 74.1  *3.87* | 62.2  *3.79, 3.89* |
| →3,4)-α-d-Man*p*-(1→  **С** | 103.4  *5.08* | 67.6  *4.32* | 73.1  *4.02* | 74.1  *3.94* | 73.9  *4.03* | 61.6  *3.81, 3.96* |
| α-l-6dTal*p*-(1 →  **D** | 98.1  *5.07* | 71.3  *3.89* | 66.7  *4.07* | 73.6  *3.77* | 68.5  *4.62* | 16.6  *1.26* |
